# Effects of Angiotensin-I-Converting Enzyme (ACE) Mutations Associated with Alzheimer’s Disease on Blood ACE Phenotype

**DOI:** 10.3390/biomedicines12102410

**Published:** 2024-10-21

**Authors:** Olga V. Kryukova, Igor O. Islanov, Elena V. Zaklyazminskaya, Dmitry O. Korostin, Vera A. Belova, Valery V. Cheranev, Zhanna A. Repinskaia, Svetlana A. Tonevitskaya, Pavel A. Petukhov, Steven M. Dudek, Olga A. Kost, Denis V. Rebrikov, Sergei M. Danilov

**Affiliations:** 1 Faculty of Chemistry, M.V. Lomonosov Moscow University, 119991 Moscow, Russia; so.b11onde@gmail.com (O.V.K.); kost-o@mail.ru (O.A.K.); 2 Medical Genetics Department, Petrovsky National Research Centre of Surgery, 117418 Moscow, Russia; bioing.bioinf@yandex.ru (I.O.I.); helenzak@gmail.com (E.V.Z.); 3 Center for Precision Genome Editing and Genetic Technologies for Biomedicine, Pirogov Russian National Research Medical University, 117997 Moscow, Russia; d.korostin@gmail.com (D.O.K.); verusik.belova@gmail.com (V.A.B.); ferondreamer911@gmail.com (V.V.C.); repinskaia@gmai.com (Z.A.R.); ncagip4@gmail.com (D.V.R.); 4 Faculty of Biology and Biotechnology, National Research University Higher School of Economics, 117418 Moscow, Russia; stonevitskaya@hse.ru; 5 Department of Pharmaceutical Sciences, College of Pharmacy, University of Illinois, Chicago, IL 60612, USA; pap4@uic.edu; 6 Department of Medicine, Division of Pulmonary, Critical Care, Sleep and Allergy, University of Illinois, Chicago, IL 60612, USA; sdudek@uic.edu

**Keywords:** angiotensin-I-converting enzyme, mutations, conformational changes, blood ACE, screening, Alzheimer’s disease

## Abstract

Backgrounds. Our recent analysis of 1200+ existing missense ACE mutations revealed that 400+ mutations are damaging and led us to hypothesize that carriers of heterozygous loss-of-function (LoF) ACE mutations (which result in low ACE levels) could be at risk for the development of late-onset Alzheimer’s disease (AD). Methods. Here, we quantified blood ACE levels in EDTA plasma from 41 subjects with 10 different heterozygous ACE mutations, as well as 33 controls, and estimated the effect of these mutations on ACE phenotype using a set of mAbs to ACE and two ACE substrates. Results. We found that relatively frequent (~1%) AD-associated ACE mutations in the N domain of ACE, Y215C, and G325R are truly damaging and likely transport-deficient, with the ACE levels in plasma at only ~50% of controls. Another AD-associated ACE mutation, R1250Q, in the cytoplasmic tail, did not cause a decrease in ACE and likely did not affect surface ACE expression. We have also developed a method to identify patients with anti-catalytic mutations in the N domain. These mutations may result in reduced degradation of amyloid beta peptide Aβ42, an important component for amyloid deposition. Consequently, these could pose a risk factor for the development of AD. Conclusions. Therefore, a systematic analysis of blood ACE levels in patients with all ACE mutations has the potential to identify individuals at an increased risk of late-onset AD. These individuals may benefit from future preventive or therapeutic interventions involving a combination of chemical and pharmacological chaperones, as well as proteasome inhibitors, aiming to enhance ACE protein traffic. This approach has been previously demonstrated in our cell model of the transport-deficient ACE mutation Q1069R.

## 1. Introduction

Alzheimer’s disease (AD) is the most common cause of dementia and a growing global health concern, afflicting about 35 million people worldwide. Extracellular β-amyloid peptide (Aβ42) deposition in senile plaques [1] is considered to initiate a cascade of events leading to AD development. Multiple genes have been linked to AD risk, but AD etiology remains incompletely understood. Rare cases of early-onset familial AD are associated with mutations in amyloid precursor protein *APP* [2] and presenilins (*PS1* and *PS2*) [3], while late-onset AD is multifactorial and associated with mutations and polymorphic alleles in *APOE*, as well as up to 40 other different genetic risk loci [4,5,6].

One such gene associated with AD risk is the gene for angiotensin-converting enzyme (ACE, CD143, EC 3.4.15.1) [6,7,8]. ACE is a Zn^2+^-dipeptidyl carboxypeptidase that metabolizes a number of metabolically active peptides and is a central component of the renin–angiotensin system. It plays key roles in blood pressure control, the development of vascular pathology, and innate immunity (reviewed in [9,10]). The mechanism responsible for the association of the ACE gene with AD may be direct and straightforward, namely via the hydrolysis of amyloid peptides by ACE [11,12,13,14]. Specifically, the active N domain center of ACE degrades in vitro the major Aβ component of the plaques, Aβ42 [14]. Therefore, less ACE may result in increased Aβ42 levels and a higher risk of AD. We hypothesize that carrying damaging heterozygous ACE mutations will be the primary cause of patients’ low ACE expression [15]. Furthermore, genetic association studies by Kehoe’s group on Aβ peptide in cerebrospinal fluid (CSF) and ACE-risk related haplotypes [16] have provided strong supportive evidence that gene variations in ACE were likely important to Aβ peptide degradation in people.

Recently, we analyzed multiple existing sequencing databases for ACE mutations and found 1200+ ACE mutations, among which 400+ were potentially damaging [15]. The high predicted frequency of damaging ACE mutations (about 3% in the general population) suggests that a significant number of individuals could therefore have very low ACE activity. These data inspired the hypothesis that carriers of **heterozygous** loss-of-function (LoF) ACE mutations may be at increased risk for Alzheimer’s because they have only one functional allele and thus are expected to have only half the normal level of ACE activity. While most homozygous carriers of LoF ACE mutations die in utero [17,18], heterozygous ones may function quite normally, except for the enhanced risk of Aβ42 accumulation and, as a result, late-onset AD development [15].

The aim of this current work was to establish an approach to quantify blood ACE levels in **EDTA plasma** from patients with different missense ACE mutations (the only source of blood ACE in sequencing facilities) and estimate the effect of these ACE mutations (changing amino acid residues) on different characteristics of ACE phenotype (including catalytic properties) using a set of mAbs to ACE and two ACE substrates.

We found in this study that the most common (about 1%) AD-associated ACE mutation, Y215C [6], is truly damaging and likely transport-deficient, because most of the carriers of these mutations possessed only about half as much ACE in the blood as control subjects without ACE mutations. Those with another relatively common ACE mutation, R1250Q, which is also associated with AD [19], had practically normal blood ACE levels, indicating that the mechanism of association of this ACE mutation with AD may be different from that for ACE mutation Y215C (which could be simply due to a decrease in surface ACE expression). We also established an approach to detect patients with damaging anti-catalytic mutations in the N domain active center, which could be AD-associated, because Aβ42 is preferentially degraded by this domain [14].

This analysis’s key finding is identifying subjects carrying **transport-deficient** ACE mutations, resulting in low ACE levels, who could benefit from preventive or therapeutic interventions. These interventions may involve a combination of chemical and pharmacological agents, such as centrally acting ACE inhibitors, along with chaperones and proteasome inhibitors, aimed at restoring impaired surface ACE expression. This approach has been previously successful in addressing another transport-deficient ACE mutation (Q1069R) known to cause renal tubular dysgenesis (RTD) [20].

## 2. Materials and Methods

### 2.1. Study Participants

The collection of human blood samples was approved by the Ethics Committee of the Kulakov National Medical Research Center for Obstetrics, Gynecology and Perinatology (protocol No. 9 from 22 October 2020). All corresponding procedures were carried out in accordance with institutional guidelines and the Code of Ethics of the World Medical Association (Declaration of Helsinki). All subjects provided written informed consent for sample collection, subsequent analysis, and publication thereof. The parents of the newborns gave written informed consent for the use of any data for scientific purposes. The whole study included blood samples from 35 adults, including 17 with missense ACE mutations and 18 controls, and blood samples from 39 newborns, including 24 with missense ACE mutations and 15 controls. All available patient samples were analyzed without any exclusions.

### 2.2. Whole Exome Sequencing

Genomic DNA isolation, quality assessment, DNA library preparation, further enrichment according to the “RSMU exome” protocol, and sequencing were performed as recently described [21] with SureSelect Human All Exon v7 baits (Agilent Technologies, Santa Clara, CA, USA). Enriched DNA libraries were sequenced on DNBSEQ G-400 (MGI Tech, Shenzhen, China) in PE100 mode to target mean coverage above 100×. The quality control of the obtained paired fastq files was performed by FastQC v0.11.9 [22]. Based on the quality metrics, the fastq files were trimmed using BBDuk by BBMap: https://github.com/BioInfoTools/BBMap (accessed on 8 September 2024). Reads were aligned to the indexed reference genome GRCh37 using bwa-mem2. SAM files were converted into BAM files and sorted using SAMtools v1.10 to check the percentage of the aligned reads [23]. Duplicates in the resulting bam files were marked using Picard MarkDuplicates v2.22.4:Broad Institute GitHub: Picard. URL: https://broadinstitute.github.io/picard (accessed on 8 September 2024) and were excluded from further analysis. For samples that passed quality control (with a target coverage width 10× ≥ 95%), single-nucleotide variants (SNVs) and indels were called using bcftoolsmpileup [24] and DeepVariant [25]. Subsequently, vcf files were obtained for each sample. After variant calling, vcf files were normalized using vtnormalize [26] and filtered by target regions extended by ±100 base pairs from each end. Variant calling data were annotated with InterVar [27].

### 2.3. Chemicals

ACE substrates benzyloxycarbonyl-L-phenylalanyl-L-histidyl-L-leucine (ZPHL) and hippuryl-L-histidyl-L-leucine (HHL) were purchased from Bachem Bioscience Inc. (King of Prussia, PA, USA) and Sigma (St. Louis, MO, USA), respectively. Other reagents (unless otherwise indicated) were obtained from Sigma (St. Louis, MO, USA).

### 2.4. Antibodies

The antibodies to human ACE used in this study recognize native conformations of the N and C domains of human ACE, as described previously [28,29,30].

### 2.5. ACE Activity Assay

ACE activity was measured using a fluorimetric assay with two ACE substrates: 2 mM ZPHL or 5 mM HHL [31,32]. The parameter ZPHL/HHL ratio was calculated as the ratio of the rates of the hydrolysis of ZPHL and HHL by the definite ACE sample [33].

### 2.6. Immunological Characterization of the Blood ACE

To quantify the amount of immunoreactive ACE protein in EDTA plasma, we applied an immunoassay in which native ACE from plasma samples was captured by anti-ACE mAbs recognizing conformational epitopes on the surface of ACE molecules. Microtiter (96-well) plates (Corning, Corning, NY, USA) were coated with anti-ACE mAbs via goat anti-mouse IgG (Invitrogen, Rockford, IL, USA, or IMTEK, Moscow, Russia) bridge and incubated with plasma samples that had been diluted 10 times. Next, after washing away the unbound ACE (together with EDTA and possible ACE inhibitors), precipitated ACE activity was quantified directly in the wells of the microtiter plates fluorometrically with ZPHL or HHL as substrates [31,32]. Conformational fingerprinting of blood ACE was performed as described earlier using a set of mAbs to different epitopes of ACE [28,29].

### 2.7. Statistical Analysis

Parameters characterizing ACE phenotype were determined as mean ± SD from at least 3 independent experiments with duplicates. Significance was analyzed using the Mann–Whitney test. Predictions and scores to account for evolutionary conservation and structural features were performed using the PolyPhen-2 engine [34].

### 2.8. Localization of AD-Associated ACE Mutations on ACE Globule

**Figure 1:**
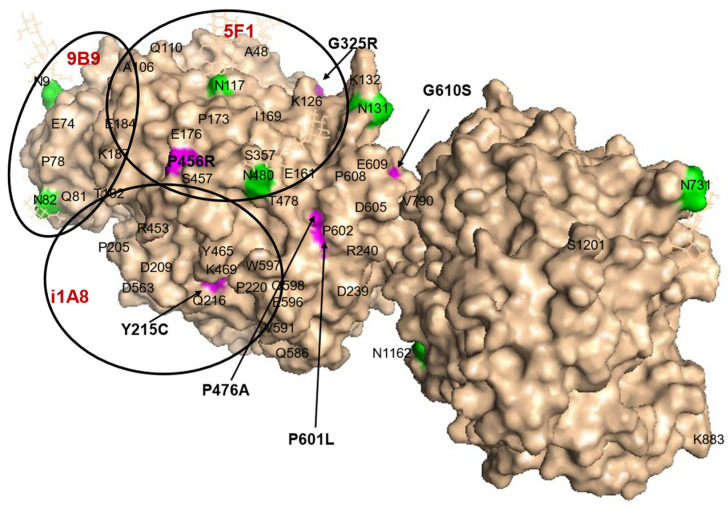
Localization of relevant ACE mutations in the N domain of ACE. The position of each ACE mutation is shown on the Cryo-EM structure of the truncated (1–1201) human somatic ACE (PDB 7Q3Y) [35] using molecular surface representation. Key amino acids are denoted using somatic ACE numbering. The surface is colored light beige, with specific amino acid residues colored as follows: ACE mutations are highlighted in magenta and additionally marked by arrows; Asn as putative glycosylation sites are highlighted in green; the last visible residue in the C-terminal end of this truncated somatic ACE is marked with its number, S1201. The epitopes for several mAbs to the N domain (9B9, 5F1, i1A8) are shown as black circles for orientation with a diameter of 30 Å, which corresponds to 700 Å^2^ of the area covered by each listed mAb.

The coordinates of the X-Ray model of the human somatic ACE were downloaded from the PDB accession #7Q3Y for Figures 1 and 6, and Figure S2 [35], and the coordinates for the truncated N domain dimer were downloaded from the PDB accession #3NXQ for Figure 4 [36]. The hydrogen atoms were added, and the resulting models were rendered in PYMOL. For Figure 7, the structure of the transmembrane domain was obtained using the homology module in Molecular Operating Environment (MOE, www.chemcomp.com, accessed on 8 September 2024) [15].

## 3. Results and Discussion

### 3.1. Quantification of Blood ACE in Carriers of ACE Mutations

We have previously established an approach for the characterization of ACE in the blood (“blood ACE phenotyping”) which includes the measurement of ACE activity, quantification of immunoreactive ACE protein, and detection of a range of conformational changes in blood ACE using a set of mAbs to ACE [28,29,32]. Unfortunately, most sequencing facilities operate only with EDTA-containing plasma samples. This makes it impossible to directly measure ACE activity due to the EDTA-mediated extraction of zinc ion from the active centers of the enzyme. However, it is possible to use ACE mAbs to precipitate the enzyme from EDTA-containing plasma, and then multiple ACE substrates can be used to characterize various ACE mutations in detail from these EDTA-treated plasma samples.

First, we determined plasma ACE protein levels (as an estimate of ACE activity) in EDTA plasma samples from 17 adult carriers of different ACE mutations and 18 controls using a set of multiple mAbs to ACE [30]. We also determined ACE levels in blood samples obtained from newborns (twenty-four carriers of eight different ACE mutations and fifteen controls) using only one mAb 9B9 due to the very limited volume of these plasma samples. Altogether, we analyzed blood samples from 41 carriers of 10 different ACE mutations. Eight of these mutations are in the N domain of the ACE protein, one mutation (L18ins) appears to be in the signal peptide region (which is cleaved during maturation), and one mutation (R1250W) is located in the cytoplasmic tail [15,19]. Note that none of these samples contained mutations in the C domain of the ACE protein.

The molecular model shown in Figure 1 illustrates the locations of six visible ACE mutations in the N domain (marked by arrows and magenta color). Another mutation (R532W) is located on the opposite side of the protein structure, while the Q259R mutation is located inside the globule and therefore is not visible.

**Figure 2:**
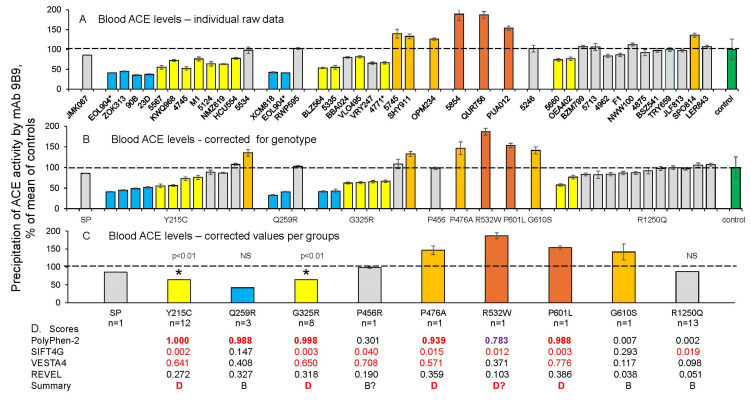
Quantification of blood ACE levels in carriers of ACE mutations. Blood ACE protein was precipitated from EDTA plasma by mAb 9B9 (which binds to an epitope on the N domain of ACE), and its activity was quantified fluorometrically using ZPHL as a substrate. (**A**) Immunoreactive ACE protein was quantified in plasma samples obtained from 41 carriers of 10 different ACE mutations. Asterisk indicates that ACE from subject 4771 had two mutations. (**B**) Plasma ACE levels adjusted according to the donor’s genotype for the I/D polymorphism [37,38]. (**C**) ACE levels (from (**B**)) were calculated for each group of subjects with the specified ACE mutation; “*n*” = the number of donors in each group. For carriers of the Y215C, G325R, Q259R, and R1250Q mutations, corresponding median values were calculated and significance analyzed using the Mann–Whitney U test. ACE levels for the other mutations in which only a single subject was available for sampling were presented as the means +/− standard deviations of several independent assessments of those individual samples. Data were expressed as % of ACE levels compared to the corresponding value for the pooled control plasma samples from subjects without ACE mutations (green bars). Orange and brown bars indicate samples with ACE levels higher than 120% and 150% of those of controls, respectively. Yellow and blue bars indicate samples with ACE levels lower than 80% and 50% of those of controls, respectively. Grey bars-values between 80% and 120% from control values. (**D**) Predictions of the potential damaging effects of nine mutations on the ACE protein using four different predictive tools, derived from Table S1 [15]. Values shown in red are predicted to be damaging by the listed predictive engine; purple-probably damaging, values in black are predicted to be benign. * *p* < 0.01.

Shown in Figure 2A are ACE blood levels for all tested carriers of ACE mutations, as determined using mAb 9B9. Of note, a review of multiple existing sequencing databases has identified >1200 ACE mutations. At least 400 of these mutations are predicted to be potentially damaging in terms of possible alterations in ACE expression or function. Therefore, the ratio of ACE gene mutation importance in this group is approximately one third.

We previously reported that a patient with the **R532W** (rs4314) ACE mutation exhibited a highly elevated ACE plasma level (approximately fivefold). This elevation was attributed to a significant increase in ACE shedding, as the R532W mutant lacked the ability to bind to bilirubin or lysozyme. Consequently, the mutant protein could not adopt the correct conformation on the cellular membrane [39]. In the current study, we report data for another subject with the R532W ACE mutation (patient QUR756), who also exhibits elevated levels of ACE in plasma (Figure 2A,B), but not to the same extent as in [39]. This difference in blood ACE levels between these two subjects with the R532W (rs4314) mutation may be explained in part by the fact that rs4314 results in two possible amino acid substitutions in the ACE protein—R532W and R532G. Another possible reason is that differential genomic imprinting may occur in these two patients with the R532W (rs4314) ACE mutation.

In addition, we found that carriers of two AD-associated mutations in the N domain of ACE, **Y215C** [6] and **G325R** [8], were characterized by low ACE levels (Figure 2). These mutations are fairly common, comprising approximately 1% of the population identified to date. Thus, these mutations can be considered damaging (confirming the previously reported prediction by PolyPhen-2 score (Table S2 in [15]), and likely transport-deficient, because the blood ACE levels in samples from heterozygous carriers of these mutations were about 50% of those of controls (Figure 2A,B). It is worth noting that the positions of these damaging mutations are far apart on the structure of the ACE N domain (Figure 1).

In contrast, blood ACE levels were essentially normal in carriers of another frequent AD-associated ACE mutation, **R1250Q** (Figure 2A,B), which is located in the cytoplasmic tail of ACE. Therefore, it is likely that the mechanism of association of this mutation with Alzheimer’s disease [19] is different than for ACE mutations Y215C and G325R.

No consistent pattern was noted in the three subjects with mutation **Q259R**. One possessed a normal ACE level, a second demonstrated a ~50% reduction in blood ACE (suggesting a possible transport-deficient effect), while a third Q259R carrier also had the damaging Y215C mutation, which is a possible explanation for the low ACE level observed in this subject. Blood ACE levels were substantially elevated for several other ACE mutations: P476A, R532W, P601L, and G610S (Figure 2A,B).

Accurate estimation of the impact of ACE mutations on blood ACE levels for any individual requires knowledge of the genotype for the ACE I/D polymorphism, which is well known to influence ACE blood and tissue levels [40,41,42,43]. Blood ACE levels in carriers of the DD genotype are 66% higher than in carriers of the II genotype [31,44]. Therefore, all values of blood ACE levels in carriers of the 10 mutations are expressed both as % of the mean found in control samples without ACE mutations (Figure 2A), as well as the results of adjustment for the ACE genotype in each individual, as described in [37,38]. These adjusted results are presented in Figure 2B, demonstrating both the influence of each mutation on blood ACE levels and inter-individual differences in these values for carriers of the same mutation.

The effects of each mutation on blood ACE levels as a group are also presented in Figure 2C. The damaging effects of Y215C on blood ACE levels were calculated as a median from the ACE level values of the 12 carriers of this mutation. Similarly, the damaging effects of G325R were calculated as a median from the ACE level values of the eight carriers of this mutation, and the results were highly statistically significant (Figure 2C). The calculated median value for the 13 carriers of R1250Q confirms that this mutation does not influence the ACE level, while the results obtained for Q259R were statistically insignificant. Note that only one blood sample was available for the other six mutations, and therefore, the putative effects of these mutations (Figure 2 and Figure S1) on blood ACE levels should be considered as preliminary estimates (Figure 2C). Nevertheless, we compared the predictive accuracy of the potential damaging effects of nine mutations on ACE protein using four different predictive tools (Figure 2D). This comparison was based upon an analysis of evolutionary, population genetic, and protein 3D structural constraints and generally demonstrated the accuracy of these predictions, especially when using the PolyPhen-2 score.

Nevertheless, more detailed characterization of some of these mutations may be clinically and diagnostically important for personalized medicine. This potential is best exemplified by the twelve carriers of ACE mutation Y215C and the eight carriers of G325R that have been identified and analyzed. In the eight carriers of the Y215C mutation, blood ACE levels were dramatically decreased (shown as blue and yellow bars in Figure 2B), indicating that this may be a transport-deficient ACE mutation. Similarly, ACE levels were dramatically decreased in the blood of the six carriers of G325R (also shown as blue and yellow bars on Figure 2B). These results are consistent with our previous report of another transport-deficient ACE mutation, Q1069R, characterized by ACE levels that were half those of controls in heterozygous parents and practically absent blood ACE in a homozygous proband with renal tubular dysgenesis [20].

We recently demonstrated that transfection of HEK cells with a DNA construct carrying the Y215C ACE mutant resulted in six to ten times less surface ACE expression than observed for WT ACE (Danilov, 2024 unpublished results), confirming our hypothesis that Y215C is a transport-deficient mutation. However, in the current study, three carriers of the Y215C mutation and one carrier of G325R have normal blood ACE levels. One person from each group even had increased ACE levels (grey and orange bars on Figure 2B). These findings may be attributed to at least two reasons: (1) significantly increased shedding of ACE produced by a gain-of-function mutation in the still unidentified ACE secretase, or due to mutations in the genes of ACE-binding proteins, such as albumin, lysozyme, and several others, which could increase the shedding of ACE and thus increase blood ACE levels; (2) a modifier gene mutation dramatically increasing surface ACE expression and thus masking the effects of the damaging Y215C ACE mutation. Analogous protecting mutations (*RELN* (H3447R) and *APOECh* (R136S)) were recently reported in carriers of *PSEN-1* E280A mutations, which delayed the development of mild cognitive impairment and dementia in carriers of this *PSEN1* mutation for about 20 years [45,46].

**Figure 3:**
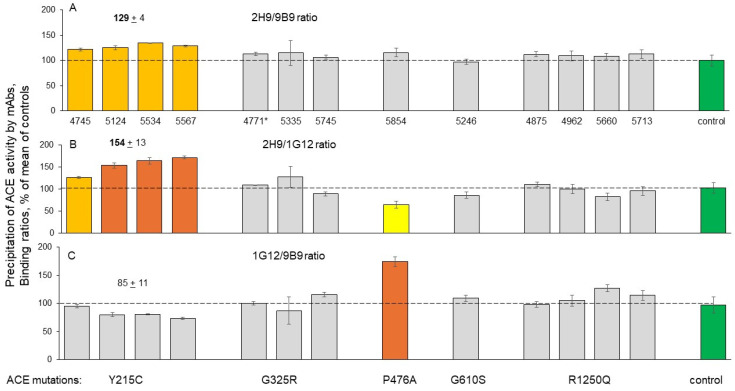
ACE precipitation by mAbs from the EDTA plasma of carriers of ACE mutations. Blood ACE protein was precipitated using two mAbs targeting the N domain (9B9 and 1G12) and mAb2H9, targeting the C domain. Precipitated ACE activity was quantified as in Figure 2. (**A**) 2H9/9B9 binding ratio; (**B**) 2H9/1G12 binding ratio; (**C**) 1G12/9B9 binding ratio. The standard deviations (SDs) for precipitated ACE activity for all three mAbs did not exceed 10%; therefore, their ratios were presented as mean (without individual SD). Bar coloring is the same as in Figure 2.

Blood ACE phenotyping provides a method for not only identifying damaging ACE mutations but also for suggesting putative mechanisms by which these mutations could contribute to Alzheimer’s disease development. For example, a low level of ACE in the blood could be due to decreased surface ACE expression caused by a mutation that produces a transport deficiency (such as the impaired trafficking of mutant ACE to the cell surface caused by Y215C and G325R). In this regard, it is important that Y215C mutant ACE (detailed localization is shown in Figure S2) can be detected (and quantified) in the blood using simultaneous ACE precipitation by two mAbs, one targeting the N domain (9B9 or 1G12) and another the C domain (mAb 2H9) of the enzyme. The results of this approach are presented in Figure 3.

The calculated 2H9/9B9 and 2H9/1G12 binding ratios effectively distinguish ACE with the damaging Y215C mutation from control ACE without any mutation, or from ACE with the other mutations characterized in this study, including the damaging G325R mutation (Figure 3). Both ratios, 2H9/9B9 (Figure 3A) and 2H9/1G12 (Figure 3B), are significantly higher for Y215C ACE than for the other ACE variants. This approach may have clinical utility in the future as an objective method to measure mutant ACE in the blood during therapeutic attempts to compensate for abnormal trafficking of the protein. In addition, Y215C mutant ACE is characterized by decreased values for the 9B9/i1A8 and 1G12/5F1 ratios of mAbs binding (Figure S1).

**Figure 4:**
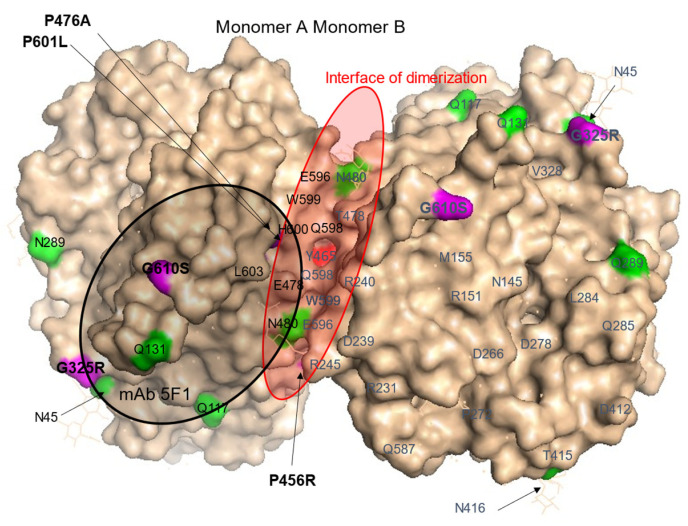
Localization of P476A, P601L, and G610S mutations in the N domain of ACE. Shown is a molecular surface presentation of the crystal structure for the N domain dimer of human ACE, where seven potential Asn glycosylation sites were substituted by Gln residues (PDB 3NXQ). Key amino acids are denoted using somatic ACE numbering. The surface is indicated by light beige, with specific amino acid residues colored as follows: Asn or Asn substituted by Gln in some putative glycosylation sites [36] are highlighted in green; ACE mutations (P476A, P601L, and G610S) are highlighted in magenta. The epitope for mAb5F1 in the N domain was used to test blood samples with these ACE mutations and is marked with a black circle. The interface of dimerization of the N domain [47,48] is shown as a red ellipse, with Y465 marked in bright red.

The subject with the **P476A** ACE mutation demonstrated significantly increased blood ACE levels, estimated with not only mAb 9B9 (Figure 2A,B) but also using five other mAbs to the N domain of ACE (1G12, 2H9, i1A8, 2D1, 2D7) and one mAb, 2H9, with its epitope in the C domain but quite close to the N domain. However, the ACE level in this subject corresponded to the normal range when estimated with the mAb 5F1 to be in the N domain (Figure S3A). Localization of the P476A mutation in the N domain dimer and, more specifically, at the interface of ACE dimerization (Figure 4) suggests that the observed twofold increase in blood ACE level could be due to altered dimerization leading to more shedding of this mutant ACE, similar to that observed in carriers of Y465D [47].

Thus, these results support previous predictions about the putative effects of mutations located at the interface of dimerization on ACE shedding [47,48]. Note that subjects with the Y465D mutation and dramatically increased (fivefold) blood ACE levels also demonstrated decreased blood ACE precipitation by mAb 5F1 [47]. The elevated 1G12/5F1 binding ratio (with ZPHL as a substrate) could be a marker of P476A ACE mutation (218% compared to control, *p* < 0.05) (Figure S1D).

ACE mutation **G610S** in an adult subject and ACE mutation P601L in a newborn patient (PUA012) are both located at the interface of N domain dimerization (Figure 4, see also [48]). The carriers of these mutations demonstrated increased blood ACE levels in comparison to control (Figure 2A,B, Figure S1 and Figure S3). The blood sample from newborn PUA012 was very small, so we were unable to determine the binding of different mAbs to ACE with P601L mutation. However, the G610S ACE mutation could be detected using the 1G12/5F1 binding ratio (with HHL as a substrate), suggesting that this ratio may be a marker for the G610S ACE mutation (158% from control, *p*< 0.05, calculated from Figure S1D).

### 3.2. Detection of Catalytic Abnormalities of Mutant ACEs Using EDTA Plasma Samples

In addition to mutations that reduce ACE surface expression (such as Y215C and G325R), other types of mutations could alter ACE structure and function and potentially contribute to the development of Alzheimer’s disease. Since the peptide Ab42 is hydrolyzed in the N domain active center [16], mutations in this area of the ACE protein could decrease Ab42 hydrolysis and be a risk factor for the disease. To identify putative carriers of this type of mutation, we applied our previously established approach, which involves precipitation of native ACE by mAbs from EDTA plasma; detection of the enzyme activity with two substrates, ZPHL and HHL; and calculation of the ratio of the rates of the hydrolysis of these substrates (the ZPHL/HHL ratio) [28,49]. An increase in this ZPHL/HHL ratio indicates partial inhibition/denaturation of the C domain active center, while a decrease in the ZPHL/HHL ratio indicates the inhibition/denaturation of the N domain active center [33].

**Figure 5:**
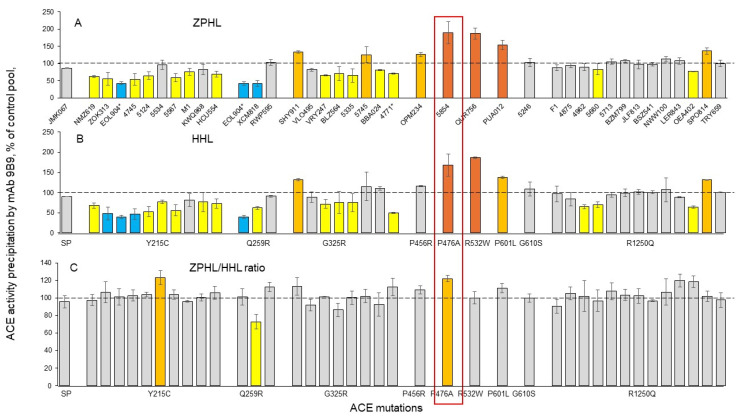
Effects of different ACE mutations on the catalytic properties of ACEs. Blood ACE protein was precipitated from EDTA plasma using mAb 9B9. Precipitated ACE activity was quantified fluorometrically as in Figure 2, using ZPHL (in (**A**)) and HHL (in (**B**)) as substrates. Data in (**C**) were expressed as a % of the ZPHL/HHL hydrolysis ratio obtained from control samples. Coloring of bars is the same as in Figure 2. Values for the P476A mutant are outlined in the orange box.

Among blood samples from the carriers of eight mutations in the N domain (and one mutation in the cytoplasmic tail, R1250Q), we identified one carrier of the **Q259R** ACE mutation with a significantly decreased ZPHL/HHL ratio (using mAb 9B9) (Figure 5).

**Figure 6:**
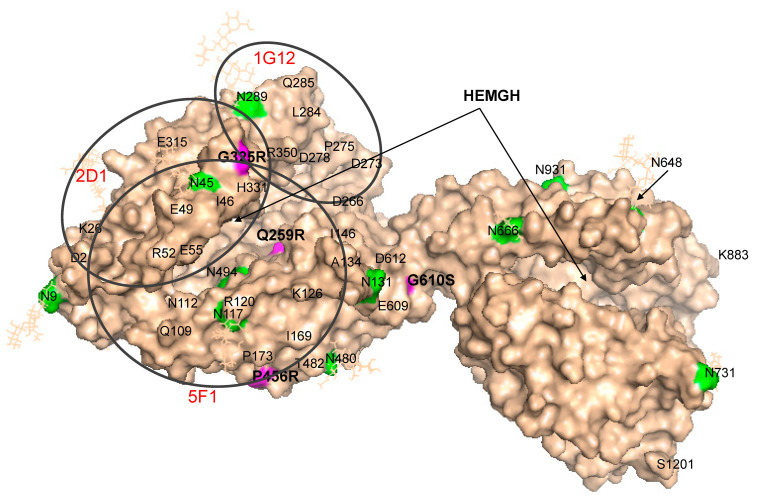
Localization of Q259R and G325R mutations in the N domain of ACE. Shown is the Cryo-EM structure of the truncated (1–1201) human somatic ACE (PDB 7Q3Y) [35] using molecular surface representation. The coloring is the same as in Figure 1. ACE mutations Q259R and G325R are highlighted with magenta. The epitopes for mAbs in the N domain (5F1/2D1, and 1G12) used to test these blood samples are outlined with black circles.

Detailed localization of the Q259R mutation on the N domain of ACE (Figure 6) clearly shows that this amino acid residue is positioned deeply inside the active center groove, very near to the catalytically important residues. The location of the Q259R mutation could lead to reduced catalytic activity of the ACE N domain and might be considered as a potential risk factor for AD.

The ZPHL/HHL ratio was significantly increased in one subject (#5534) within the Y215C mutation group and in one subject (#5854) with the P476A mutation (Figure 5C). Note that #5534 is a clear outlier, as the blood ACE level was not dramatically decreased as in other carriers of the Y215C mutation (Figure 2A,B). Careful analysis of the sequence of this patient may help identify other possible genetic reasons for the normal level of ACE.

We analyzed the ZPHL/HHL ratio for normal blood ACE precipitated by different mAbs and found that this ratio was dramatically decreased when mAbs 2D1 and 5F1 were used (Figure S4A). These results confirm the previous observation that mAb 5F1 (which has an overlapping epitope with mAb 2D1 [30]) is anticatalytic [50].

In contrast, blood from the subject with the **P476A** mutation demonstrated increased values of the ZPHL/HHL ratio when precipitated not only by mAb 9B9 but also by three weak mAbs: i1A8, 2D1, and 5F1 (Figure S4D). Thus, an increased ZPHL/HHL ratio can be considered as another marker for the P476A ACE mutation. The increased ZPHL/HHL ratio observed with the P476A mutant could be due to conformational changes in the C2^loop^ of the N domain (residues 472–498) (Figure 7 in [35] and Figure S3E), which contains the catalytically essential residues K489 and Y498 that form a critical anchor for substrate/inhibitor binding [35,51]. The position of the mutated P476 amino acid residue is shown in Figure S3E. It is likely that the Pro substitution by Ala in the P476A mutant induces significant conformational changes that may increase the catalytic activity of the N domain active center. Therefore, it seems less likely that the P476A mutation in the N domain of ACE is associated with Alzheimer’s disease. Interestingly, three other ACE mutations (P456R, P601L, and G610S) that are located in the interface of ACE dimerization (Figure 4) and characterized by the increased blood ACE levels (Figure 2) did not demonstrate an increased ZPHL/HHL ratio (Figure 5C and Figure S4). A potential explanation is that these two mutations are located outside of the C2^loop^ in the N domain (Figure S3E) and thus do not affect the catalytically essential residues K489 and Y498.

ACE with mutation **G325R** demonstrated an increased ZPHL/HHL ratio only when precipitated with mAb 2H9 to the C domain of ACE (Figure S4B). The reason for this observation is unclear. Detailed localization of the G325R mutation (shown in Figure 6) indicates that this amino acid residue is positioned on the edge of the active site cleft. This position explains the anticatalytic properties of mAbs 5F1 and 2D1 (which have this residue in their epitopes) and may block the entrance to the N domain active center groove and fix movement of the jaws of the N domain active center, necessary for hydrolysis [35,52]. Analysis of mAbs binding to carriers of the G325R ACE mutation demonstrated that the 1G12/5F1 and 2H9/5F1 ratios (but only with ZPHL as a substrate) can serve as markers for this mutation since they are decreased for carriers of the G325R mutation to 43% of the control level (*p* < 0.05).

**Figure 7:**
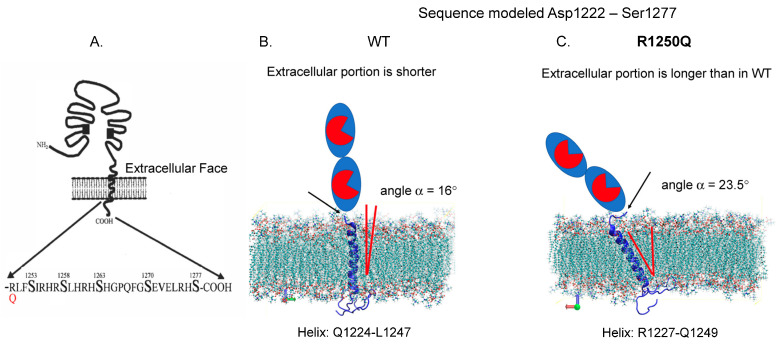
Localization of AD-associated ACE mutation R1250Q in the cytoplasmic tail. (**A**) Schema showing the localization of R1250Q ACE mutations in the cytoplasmic tail of ACE, adapted from [53]. (**B**,**C**) Molecular dynamic simulations of the transmembrane and cytoplasmic domains of ACE in POPC lipid membranes. The sequence is modeled from position Asp1222 to Ser1277. (**B**) is the WT helix span from Q1224 to L1247, and (**C**) is the R1250Q ACE mutant helix span from R1227 to Q1249. The average angles of transmembrane helices in mutant ACEs were changed in the lipid bilayers in comparison with WT ACE (from [15], with permission from publisher).

Carriers of the **R1250Q** mutation in the cytoplasmic tail (Figure 7) exhibited neither altered blood ACE levels (Figure 2) nor any changes in the ZPHL/HHL ratio when precipitated with different mAbs (Figure 5 and Figure S4F).

One hypothesis is that the reported association of this mutation with Alzheimer’s disease [19] may be caused by subtle conformational changes in the ACE molecule induced by this substitution due to crosstalk between the extracellular and cytoplasmic portions of ACE. Hence, despite its cytoplasmic location, this mutation is likely to have direct effects on the conformation of ACE at the membrane and potentially on the extent of ACE dimerization. These effects would decrease the hydrolysis of large substrates such as amyloid peptide Aβ42 [15]. Interestingly, 12 of the 13 carriers of this mutation who developed AD were women [19]. Furthermore, we observed significant differences in the conformation of urinary ACE between males and females, likely attributed to differential glycosylation patterns, particularly sialylation, in kidney ACE—the primary source of ACE in urine. The sex-specific variations in tissue ACE glycosylation identified in our study [54] may contribute to differences in disease susceptibility. It is reasonable to speculate that the R1250Q mutation could markedly disrupt Aβ42 cleavage in female carriers of this ACE mutation, while potentially exerting a different effect in males [15].

The results obtained in this study from blood ACE phenotyping in carriers of 10 different ACE mutations were added to an updated version of a table in which blood ACE levels were estimated or quantified in the carriers of 62 ACE mutations (Table S1). Excerpted data from this table (only ACE mutations with estimated ACE activity) convincingly indicate that blood ACE levels were significantly decreased in a substantial number of patients with damaging ACE mutations and Alzheimer’s disease (Table S2).

Note, however, that the present study was limited by the extremely small volumes of the plasma samples available from the tested subjects, especially from the newborns (100 ul). Therefore, we were precluded from studying the important functional and clinically relevant characteristics of mutant ACEs, such as the binding of ACE inhibitors, the hydrolysis of natural substrates for ACE (AI, bradykinin, Ac-SDKP), and especially the hydrolysis of beta-amyloid peptide Aβ42. Subsequent study requires ACE that is a hundred times purer than what is used for the assays described in this paper [14], making it impractical to perform with unprocessed EDTA plasma. Consequently, these investigations will necessitate the use of cell models and overexpression plasmids carrying various ACE mutations, as significantly more mutant ACE protein will be needed than what was available in the patient plasma samples (approximately 100 microliters per carrier of these mutations). We intend to carry out these crucial experiments in the near future.

Another potential limitation of the current study is that ACE phenotyping was performed in samples obtained from carriers of these mutations, not from AD patients. However, this work still has important relevance to AD pathophysiology by advancing understanding about the possible mechanisms by which ACE mutations that are significantly associated with AD may contribute to the development of the disease.

## 4. Conclusions

1. The ACE phenotyping method utilizes the precipitation of ACE by different mAbs in combination with the subsequent measurement of ACE activity [28,31,32,49] to quantitatively estimate the effects of these ACE mutations. It also allows for the identification of patients with transport-deficient ACE mutations that are characterized by a substantial decrease in blood ACE levels (for example, carriers of the common ACE mutations Y215C and G325R).
2. Combining the various ACE mAbs at our disposal [29,30] with multiple substrates theoretically allows for the detection of individuals with ACE mutations in the N domain active center that decrease catalytic activity. These mutations may inhibit the ability to hydrolyze N-domain-specific substrates, including Aβ42 [14], which could increase AD risk.
3. Using only EDTA-containing plasma samples for ACE phenotyping precludes the direct measurement of blood ACE activity in solution from an individual, but this approach can yield patterns and markers indicative of the effects of ACE mutations. This is achieved through the application of a set of ACE mAbs along with various ACE substrates. This approach is especially useful for transport-deficient ACE mutations. It is conceivable that future clinical trials may target carriers of these transport-deficient ACE mutations to test the effectiveness of a cocktail of chemical and pharmacological chaperones, as well as proteasome inhibitors to rescue the impaired trafficking of mutant ACEs to the cell surface. Such an approach was previously successful as a “proof of concept” in an in vitro model of a rare transport-deficient ACE mutation (Q1069R) [20].
4. To estimate the effects of such therapy in future clinical trials, it will be necessary to quantify mutant ACE levels in the blood of patients. We have established such markers for both of the transport-deficient ACE mutations identified in this study: the 2H9/1G12 binding ratio (with ZPHL as a substrate) distinguishes ACE with the Y215C mutation from normal ACE or from ACE with other tested mutations, whereas the 1G12/5F1 binding ratio (also with ZPHL as a substrate) is a marker for the G325R mutation.

## Data Availability

The data supporting the findings of this study are available within the article.

## Supplementary Materials

The following supporting information can be downloaded at: https://www.mdpi.com/article/10.3390/biomedicines12102410/s1, Figure S1: Effects of ACE mutations on mAbs binding to mutant ACEs; Figure S2: Localization of the Y215C mutation in the N domain of ACE; Figure S3. Conformational mAb fingerprinting of ACE mutations (P476A and G610S); Figure S4. Effects of mAbs and various ACE mutations on the ZPHL/HHL ratio; Table S1: Existing human ACE mutations.1234_62 (09/29/23)- Table S2: 62 ACE mutations for which blood ACE levels were estimated or measured.
