# Supplementary material for: Effects of Angiotensin-I-Converting Enzyme (ACE) Mutations Associated with Alzheimer’s Disease on Blood ACE Phenotype
Source: Biomedicines. 2024 Oct 21;12(10):2410. doi: 10.3390/biomedicines12102410 (PMC11504702; doi:10.3390/biomedicines12102410)
Supplement: Supplementary file 1 [file biomedicines-12-02410-s001.zip › biomedicines-3224268-supplementary.pdf]

SUPPLEMENTARYMATERIALS.

Effects of Angiotensin I-Converting Enzyme (ACE) mutations, associated with Alzheimer's disease, on blood ACE phenotype

Olga V. Kryukova[1], Igor O. Islanov[2], Elena V. Zaklyazminskaya[2], Dmitry O. Korostin[3], Vera A. Belova[3], Valery V. Cheranev[3], Zhanna A. Repinskaia[3], Svetlana A. Tonevitskaya[4], Pavel A. Petukhov[5], Steven M. Dudek[6], Olga A. Kost[1], Denis V. Rebrikov[3] and Sergei M. Danilov[6].

[1]Faculty of Chemistry, M.V. Lomonosov Moscow University, Moscow, Russia,
[2]Medical Genetics Department, Petrovsky National Research Centre of Surgery, Moscow, Russia,
[3]Center for Precision Genome Editing and Genetic Technologies for Biomedicine,
Pirogov Russian National Research Medical University, Moscow, Russia,
[4]Faculty of Biology and Biotechnology, National Research University Higher School of Economics, Moscow, Russia,
[5]Department of Pharmaceutical Sciences, College of Pharmacy, University of Illinois, Chicago, IL, USA,
[6]Department of Medicine, Division of Pulmonary, Critical Care, Sleep and Allergy, University of Illinois at Chicago, IL, USA.

Running Head: ACE mutations and blood ACE levels

*Corresponding author:
Sergei M. Danilov, MD, PhD
Department of Medicine,
Division of Pulmonary, Critical Care, Sleep and Allergy,
University of Illinois at Chicago
CSB 915, MC 719, 840 S. Wood Ave.
Chicago, IL60612
Phone: (708)-642-0635,
E-mail:danilov@uic.edu

Key words: Angiotensin I-converting enzyme, mutations, conformational changes, blood ACE, screening, Alzheimer's disease

Fig.S1. Effects of ACE mutations on mAbs binding to mutant ACEs. A. ACE was precipitated from the EDTA-plasma of subjects with different mutations using mAbs 9B9 and i1A8 to the N domain of ACE. Precipitated ACE activity was quantified fluorometrically using HHL as a substrate as described in Fig.2. Data are presented as the 9B9/1IA8 binding ratio expressed as % obtained from control patients without ACE mutations. **B-D.** Precipitated ACE activity was determined in the plasma of carriers of different ACE mutations as in **A,** but using mAbs 1G12 and 5F1, and expressed as (**B, C**) precipitated ACE activity (% of control samples without ACE mutations) and (**D**) as the 1G12/5F1 binding ratio for each mutant (% of control). Each value represents the mean of 3-5 independent experiments.
Coloring is the same as in Fig.2.

Figure S2. Localization of the Y215C mutation in the N domain of ACE.
Shown is the Cryo-EM structure of truncated (1-1201) human somatic ACE (PDB 7Q3Y) [35] using molecular surface representation. Key amino acids are denoted using somatic mature ACE numbering. The surface is colored light beige. Specific amino acid residues are colored as following: Asn in putative glycosylation sites are highlighted in green; ACE Y215C mutation is highlighted in magenta; the last visible residue in the C-terminal end of this truncated somatic ACE is marked by its amino acid number (1201). The epitopes for the mAbs to the N domain (9B9, 1G12, 5F1) and the C domain (2H9) that are used to analyze these blood samples are marked by 30 Å diameter black circles, which correspond to an approximately 700 Å^2 area covered by each mAb.

Fig. S3. Conformational mAb fingerprinting of ACE mutations (P476A and G610S). ACE activity in subjects with P476A and G610S ACE mutations was determined from EDTAplasma samples after precipitation by 7 different mAbs to the N and C domains of ACE as described in Fig.2.**A-B**. ACE activity determined with ZPHL as a substrate expressed as a % of control samples.

C-D. Data represent the ZPHL/HHL binding ratios expressed as % of controls.

E. Shown is the Cryo-EM structure for the residues 450-613 of human somatic ACE (PDB 7Q3Y) [35] displayed as a ribbon presentation. ACE mutations P456R, P476A, P601L and G610S are highlighted with magenta. Putative glycosylation sites (N480 and N494) are highlighted with green. The 472-498 C2-loop (as defined in [35]) is highlighted with grey. Highlighted in red are the residues K489 and Y498 within the cleft of the active center. A Zn^{2+} ion is also shown.

Previously we established the **ACE phenotyping** approach for comprehensive characterization of ACE in the blood. This approach includes not only measurement of blood ACE activity with different substrates and estimation of catalytical properties, but also quantification of immunoreactive ACE protein and conformational changes in ACE molecules using a wide set of mAbs [28,29,31,32,39,49].

Unfortunately, most sequencing facilities have access to only EDTA-plasma samples, which prevent direct measurement of ACE activity and estimation of catalytic properties of mutant ACEs. Nevertheless, using mAbs to ACE which we have generated in combination with multiple ACE substrates allows for the precipitation of ACE from EDTA-plasma samples and estimation of ACE activity. With this approach we can characterize the effects of ACE mutations in fine detail from EDTA-plasma samples.

When we performed primary estimation of the blood ACE levels in EDTA-plasma samples for different ACE mutations (Fig.2,5), we used strong, high-affinity mAbs 9B9 and 1G12 [30] for precipitation of the enzyme. Precipitated ACE activity quantified by these mAbs generally did not depend on the nature of substrates which we used (ZPHL or HHL) (not shown). However, when we perform conformational fingerprinting of mutant ACE using a wider set of mAbs, we need to consider that precipitation of ACE activity with some weak mAbs may be substrate-specific. This is possible because immobilization of some mAbs on plates can result in anti-catalytic properties toward the N or C domain active center when precipitating ACE (see below in Fig.S4). Therefore,

the preferred approach should be to use HHL for the quantification of precipitated ACE activity with mAbs to the N domain (because HHL is cleaved 9-fold more effective by the C domain active center), while ZPHL should be used for precipitation of ACE activity with mAbs to the C domain active center - discussed in [33].

As an example, the effect of ACE mutation Y215C on the 9B9/i1A8 ratio (Fig.S2A) was decreased only with HHL as a substrate, while the effect of this mutation on this ratio was absent with ZPHL as a substrate (not shown).

One of the goals of this study was to test multiple mAbs and substrates to determine a combination which will allow for the detection of mutant ACE in the blood and distinguish it from native (control) ACE. Identification of markers for ACE mutations will provide a method for monitoring changes in mutant ACE activity in the blood. Such an approach would be particularly useful for assessing the effectiveness of therapies designed to increase surface ACE expression. We hypothesize that this type of therapeutic strategy could be protective for some patients at risk for ACEdependent Alzheimer’s disease. Fig.S1B-D describes the effects of different ACE mutations on the precipitation of blood ACE activity by mAbs 1G12 and 5F1. The binding of mAb 5F1 was **increased** in ACE mutant G325R and **decreased** in ACE mutant P476A. Therefore, the 1G12/5F1 binding ratio could be a convenient marker for these two ACE mutations, G325R and P476A. These results could be especially important for carriers of the G325R ACE mutation because it is likely damaging and results in a transport-deficient ACE (Fig.2,5). Carriers of this mutation may benefit from rescue of impaired trafficking of mutant ACE to the cell surface through use of a cocktail of chaperones and protease inhibitors as we have previously described for the Q1069R ACE mutation [20].

Our results provide additional insights into the functional consequences of these mutations. The effects of the P476A mutation on ACE activity after precipitation by different mAbs (Fig.S3A) and the localization of this mutation in the ACE protein (Fig.4 and Fig.3E) combine to shed light

on the putative mechanism of action by which this mutation results in increased ACE shedding and altered catalytic properties (Fig.S3C and S3E).

Fig. S4. Effects of mAbs and various ACE mutations on the ZPHL/HHL ratio.

A. ACE was precipitated from EDTA-plasma samples of control subjects (without ACE mutations) using7 different mAbs to the N and C domains of ACE. ACE activity was quantified fluorometrically with ZPHL and HHL as substrates as described in Fig.2. ZPHL/HHL ratios are presented as % of that for mAb 9B9.

B-F. ACE activity was determined in plasma samples from carriers of different ACE mutations as in **A**. Data are expressed as ZPHL/HHL ratios for each mutant and mAb as % of control ACE activity (without ACE mutations). **B.** ACE activity results are presented individually from the plasma of patient #5534 with the Y215C mutation. Other values are presented as the mean from multiple carriers of a given mutation. **C**. G325R; **D**. P476A; **E**. G610S; **F**. R1250Q. Each value is a mean of 3-10 independent experiments. Coloring is the same as in Fig.2.

Quantification of the ZPHL/HHL ratio for carriers of these ACE mutations not only provided an explanation for catalytic changes in P476A (Fig.S3C, E), but this approach also helped to identify a marker for further study of an outlier in the Y215C group of ACE mutations (patient 5534 in Fig.2,5).

Table S1. Existing human ACE mutations.1234_62 (09/29/23) - below

Table S2. 62 ACE mutations for which blood ACE levels were estimated or measured - below.

References for Table S1 and S2: ACE Mutations.1234_62.09.29.23

1. Uematsu M, Sakamoto O, Ohura T, et al, A further case of renal tubular dysgenesis surviving the neonatal period. *Eur J Pediatr* 2009; **168**: 207–209.
2. Gribouval O, Morinière V, Pawtowski A, et al. Spectrum of mutations in the renin-angiotensin system genes in autosomal recessive renal tubular dysgenesis. *Hum Mut*. 2012; **33**: 316–326.
3. Schreiber R, Gubler M-C, Gribouval O, et al. Inherited renal tubular dysgenesis may not be universally fatal. *Pediatr Nephrol* 2010; **25**: 2531–2534.
4. Kryukova OV, Islanov IO, Zaklyazminskaya EV, et al. Effect of ACE mutations, associated with Alzheimer's disease, on blood ACE phenotype. *Biomedicines* 2024; (this study)
5. Xie X-Y, Zhao Q-H, Huang Q, et al, Genetic profiles of familial late-onset Alzheimer's disease in China: The Shanghai FLOAD Study. *Genes Dis* 2022; **9**: 1639–1649.
6. Gribouval O, Gonzales M, Neuhaus T, et al. Mutations in genes in the renin-angiotensin system are associated with autosomal recessive renal tubular dysgenesis. *Nat Genet* 2005; **37**: 964–968.
7. Sassi C, Ridge PG, Nalls MA, et al. Influence of coding variability in APP-Aβ metabolism genes in sporadic Alzheimer's disease. *PLOS One* 2016; **11:** e0150079.
8. Richer J, Daoud H, Geier P, et al. Resolution of refractory hypotension and anuria in a premature newborn with loss-of-function of ACE. *Am J Med Genet* 2015; **167A**: 1654-1658.
9. Kim SY, Kang HG, Kim EK, et al. Survival over 2 years of autosomal-recessive renal tubular dysgenesis. *Clin Kidney J* 2012; **5**: 56–58.
10. Michaud A, Acharya KR, Masuyer G, et al. Absence of cell surface expression of human ACE leads to perinatal death. *Hum Mol Genet* 2013; **23**: 1479–1491.
11. Tan H-J, Jian W-Y, Lv C, et al. Prenatal diagnosis and treatment for fetal angiotensin converting enzyme deficiency. *Prenat Diag* 2023; 1-5.
12. Kondoh T, Kawai Y, Matsumoto Y, et al. Management of a preterm infant with renal tubular dysgenesis: A case report and review of the literature. *Tohoku J Exp Med.* 2020; **252**: 9–14.

13. Persu A, Lambert M, Deinum J, et al. A Novel Splice-Site Mutation in Angiotensin I-Converting Enzyme (ACE) Gene, c.3691+1G>A (IVS25+1G>A), Causes a Dramatic Increase in Circulating ACE through Deletion of the Transmembrane Anchor. *PLoS One* 2013; **8**: e59537.

14. Nesterovitch AB, Hogarth KD, Adarichev VA, et al. Angiotensin I-converting enzyme mutation (Trp1197Stop) causes a dramatic increase in blood ACE. *PLoS One* 2009; **4**: e8282.

15. Danilov SM, Jain MS, Petukhov PA, et al. Novel ACE mutations mimicking sarcoidosis by increasing blood ACE Levels. *Transl Res* 2021; **230**: 5–20.

16. Danilov SM, Adzhubei IA, Kozuch AJ, et al. Carriers of heterozygous loss-of-function ACE mutations are at risk for Alzheimer's disease. *Biomedicines* 2024; **12**: 162.

17. Lalli M.A. et al. Exploratory data from complete genomes of familial Alzheimer's disease age-at-onset outliers. *Hum Mutat* 2012; **33**: 1630-1634.

18. Schwartzentruber J, Cooper S, Liu JZ, et al. Genome-wide meta-analysis, fine-mapping and integrative prioritization implicate new Alzheimer's disease risk genes. *Nat Genet* 2021; **53**: 392–402.

19. Samokhodskaya LM, Jain MS, Kurilova OV, et al. Phenotyping angiotensin-converting enzyme in blood: A necessary approach for precision medicine. *J Appl Lab Med* 2021; **6**: 1179–1191.

20. Rieder M, Taylor SL. Clark AG, Nickerson DA. Sequence variation in the human angiotensin-converting enzyme. *Nat Genet* 1999; **22**: 59-62.

21. Danilov SM, Wade MS, Schwager SL, et al. A novel angiotensin I-converting enzyme mutation (S333W) impairs N-domain enzymatic cleavage of the anti-fibrotic peptide, AcSDKP. *PLoS One* 2014; **9**: e88001.

22. Vincent KM, Alrajhi A, Lazier J, et al. Expanding the clinical spectrum of autosomalrecessive renal tubular dysgenesis: Two siblings with neonatal survival and review of the literature. *Mol Genet Genomic Med* 2022; **10**: e1920.

23. Danilov SM, Gordon K, Nesterovitch AB, et al. An angiotensin I-converting enzyme mutation (Y465D) causes a dramatic increase in blood ACE via Accelerated ACE shedding *PLoS One* 2011; **6:** e25952.

24. Ruf K, Wirbelauer J, Beissert A and Frieauff E. (2018) Successful treatment of severe arterial hypotension and anuria in a preterm infant with renal tubular dysgenesis—A case report. *Matern Health Neonat Perinat* 2018; **4**: 27.

25. Danilov SM, Lünsdorf H, Akinbi HT, et al. Lysozyme and bilirubin bind to ACE and regulate its conformation and shedding. *Sci Rep* 2016; **6:** 34913.

26. Nicolaou N, Pulit SL, Nijman IJ, et al. Prioritization and burden analysis of rare variants in 208 candidate genes suggest they do not play a major role in CAKUT. *Kidney Int* 2016; **89**: 476–486.

27. Ramoni RB, Himes BE, Sale MM, Furie KL, Ramoni MF. Predictive genomics of cardioembolic stroke. *Stroke* 2009; **40**: (*3 Suppl*), S67-S70.

28. Cuddy LK, Prokopenko D, Cunningham EP, et al. Aβ-accelerated neurodegeneration caused by Alzheimer's-associated ace variant R1279Q is rescued by angiotensin system inhibition in mice. *Sci Transl Med* 2020; **12**: eaaz2541.

29. Pescatello LS, Schifano ED, Ash GI, et al. Deep-targeted exon sequencing reveals renal polymorphisms associate with postexercise hypotension among African Americans. *Physiol Rep* 2016; **4**: e12992.

30. Danilov SM, Kalinin S, Chen Z, et al. Angiotensin I-converting enzyme Gln1069Arg mutation impairs trafficking to the cell surface resulting in selective denaturation of the Cdomain. *PLoS One* 2010; **5**: e10438.

31. Kramers C, Danilov SM, Deinum J, et al. Point mutation in the stalk of angiotensin-converting enzyme causes a dramatic increase in serum angiotensin-converting enzyme but no cardiovascular disease. *Circulation* 2001; **104**: 1236–1240.

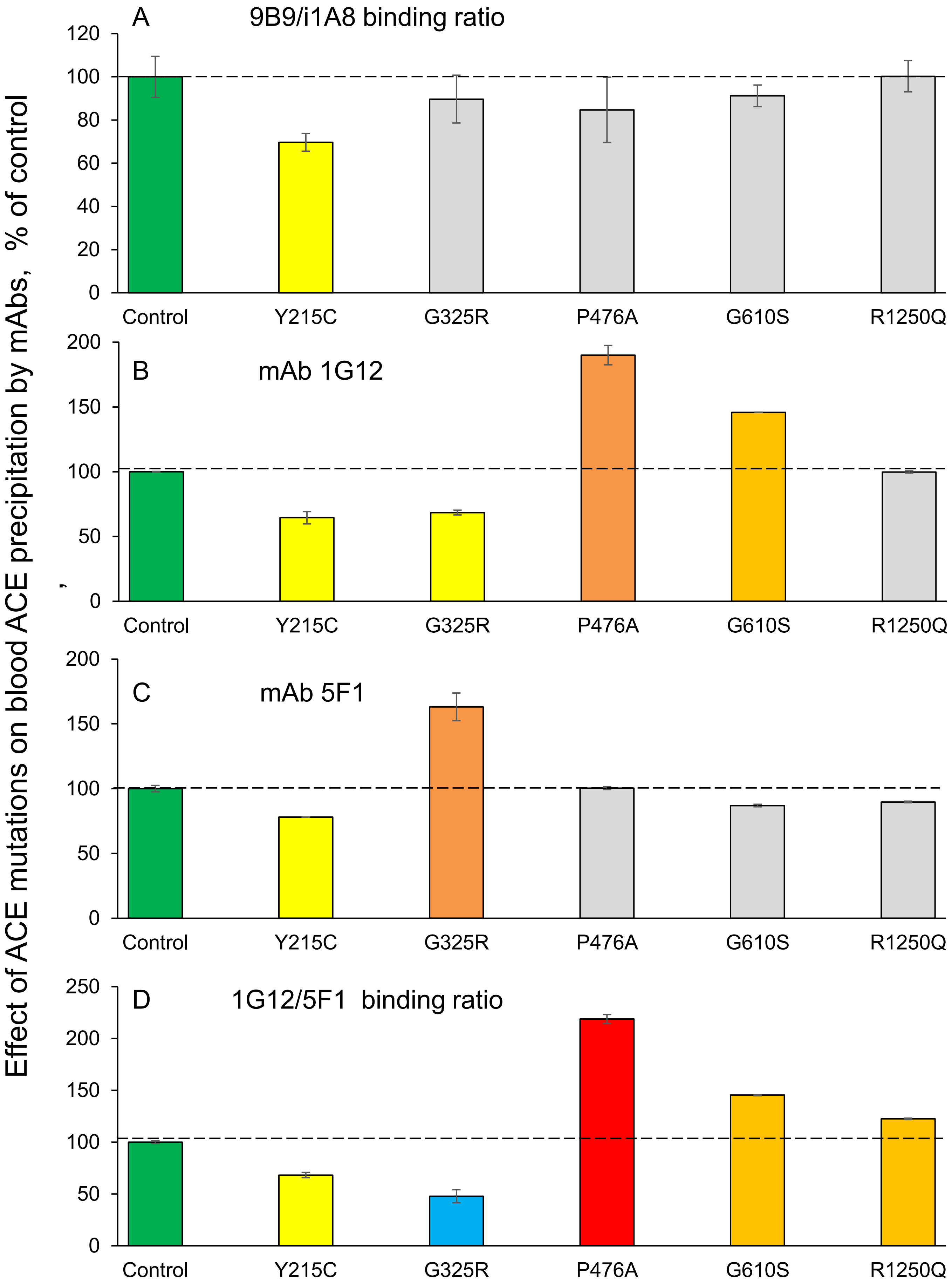
Effect of ACE mutations on blood ACE precipitation by mAbs, % of control
A
9B9/i1A8 binding ratio
0
20
40
60
80
100
120
Control
Y215C
G325R
P476A
G610S
R1250Q
B
mAb 1G12
0
50
100
150
200
C
mAb 5F1
D
1G12/5F1 binding ratio
250

Fig.S1.

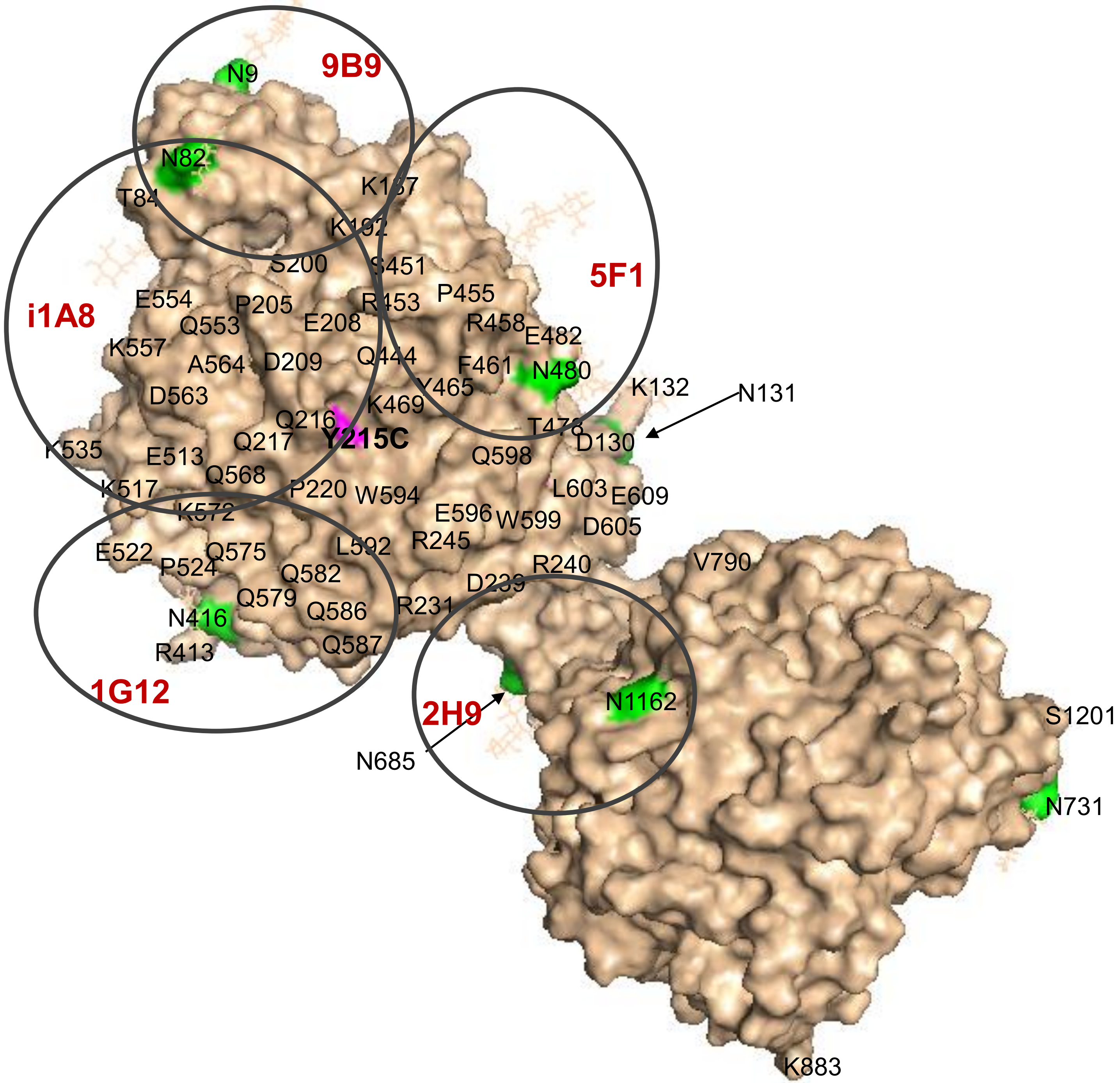
9B9
N9
N82
T84
K187
K192
S200
S451
5F1
i1A8
E554
P205
R453
P455
Q553
E208
R458
E482
K557
A564
D209
Q444
F461
N480
D563
Y465
K132
N131
K469
Q216
T478
K535
Q217
Y215C
D130
E513
Q568
Q598
K517
P220
W594
L603
E609
K572
E596
W599
D605
E522
Q575
L592
R245
P524
Q582
D239
R240
V790
Q579
Q586
R231
N416
R413
Q587
1G12
2H9
N1162
N685
S1201
N731
K883

Fig.S2.

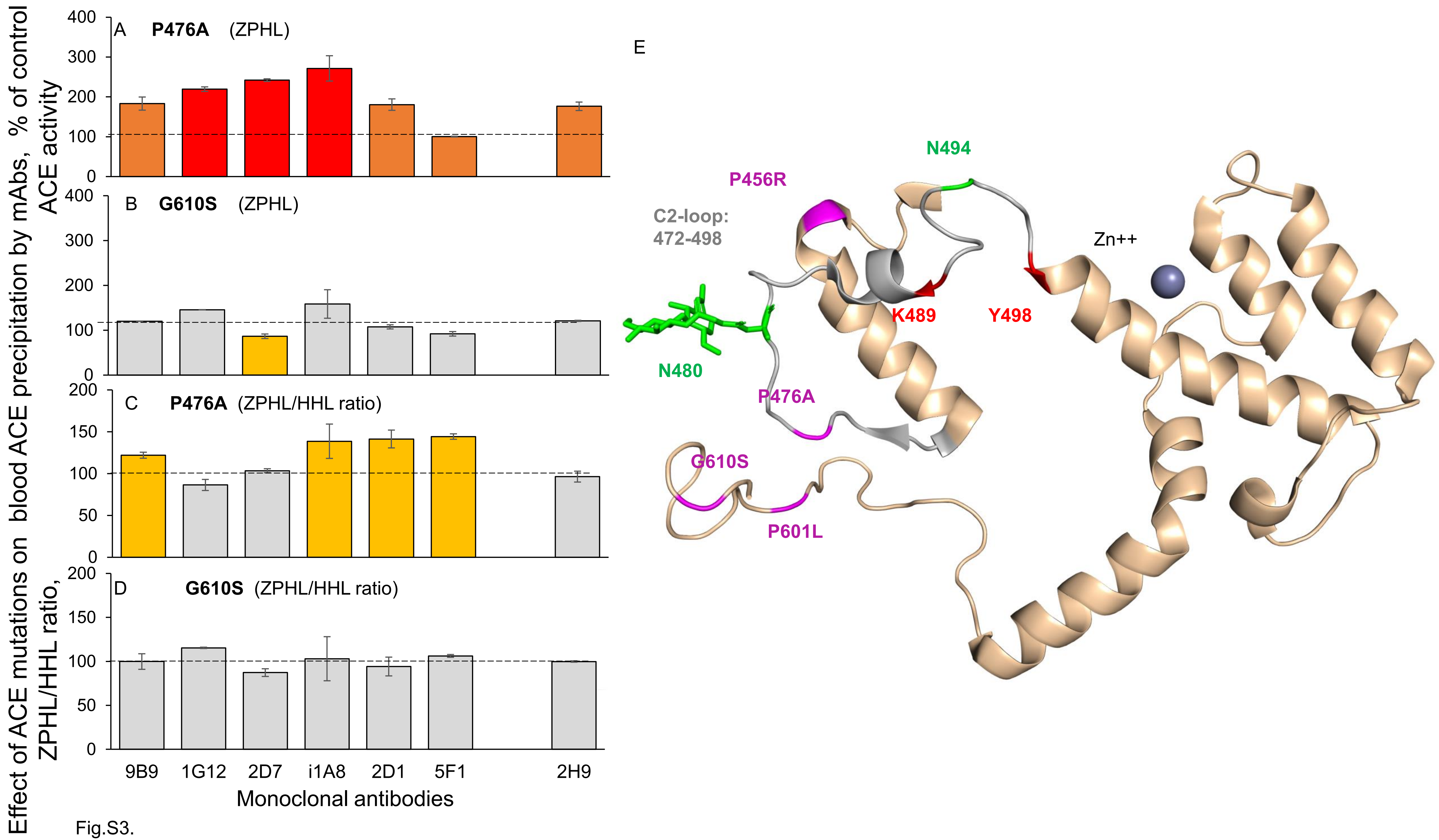
Effect of ACE mutations on blood ACE precipitation by mAbs, % of control ACE activity
ZPHL/HHL ratio,
A P476A (ZPHL)
B G610S (ZPHL)
C P476A (ZPHL/HHL ratio)
D G610S (ZPHL/HHL ratio)
9B9
1G12
2D7
i1A8
2D1
5F1
2H9
Monoclonal antibodies
E
N494
P456R
C2-loop:
472-498
Zn++
K489
Y498
N480
P476A
G610S
P601L

Fig.S3.

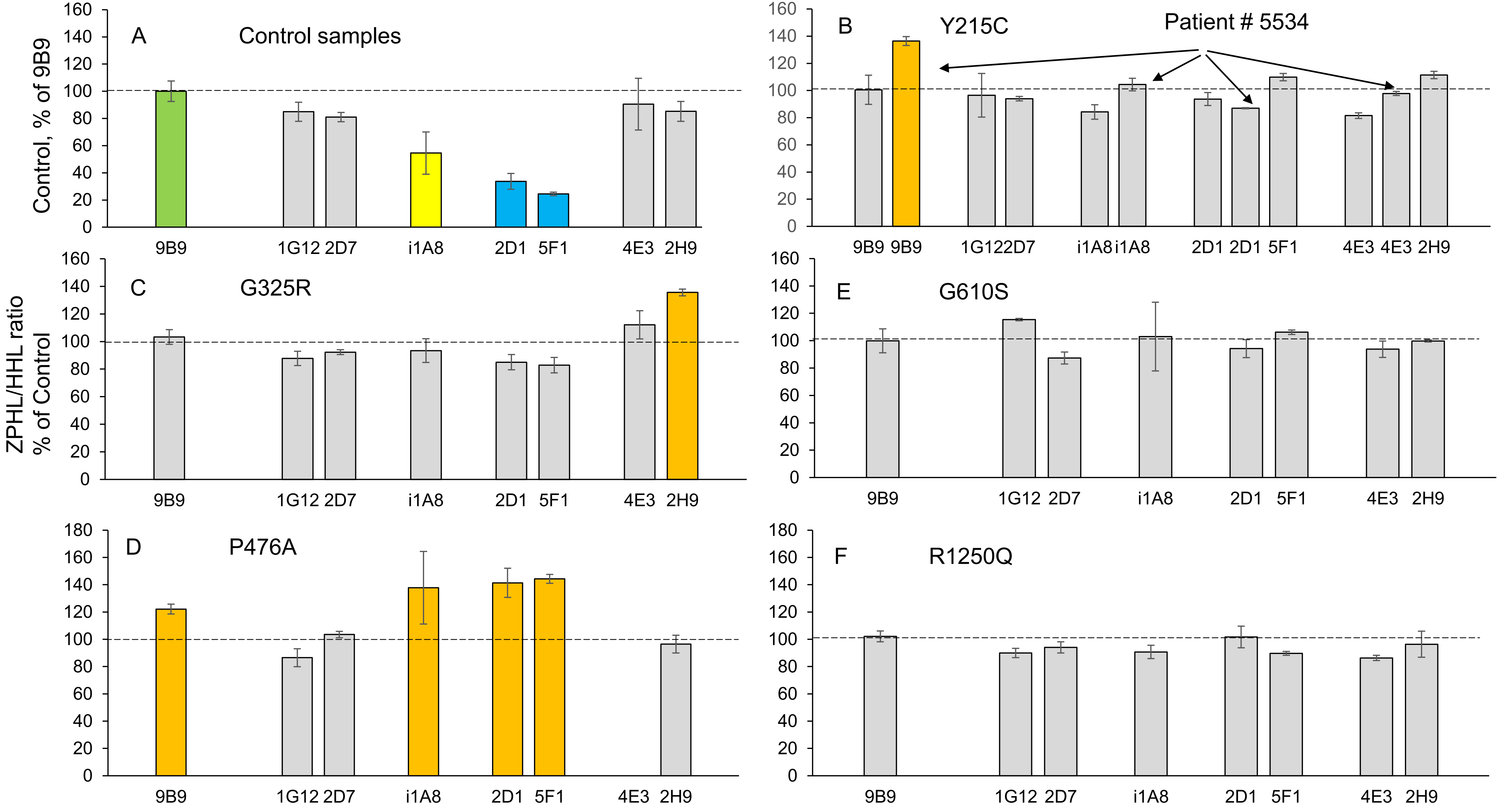
A
Control samples
Control, % of 9B9
9B9
1G12 2D7
i1A8
2D1 5F1
4E3 2H9
B
Y215C
Patient # 5534
9B9 9B9
1G122D7
i1A8 i1A8
2D1 2D1 5F1
4E3 4E3 2H9
C
G325R
ZPHL/HHL ratio
% of Control
E
G610S
D
P476A
F
R1250Q

Fig.S4.

Table S1. ACE mutations Total: **1234** Blood ACE: **62** 9/29/23

#		Amino acid position (mature protein)	Polymorphism or (*reference*)	PolyPhen-2 Score (HVAR)	Minor Allele Frequency, per 100,000	Blood ACE, % of M
**I. Damaging (elimination ?) of signal peptide (SP)**						
1	p.Met1Leu	SP	rs1262893315	0.000	0.7	
2	p.Met1Lys	SP	rs1005792910	0.016	0.7	
3	p.Met1Thr	SP		0.072		
4	p.Gly2Arg	SP	rs2049627089	**0.546**	6.0	
5	p.Gly2Glu	SP	rs558593002	0.009	**50**	
6	p.Gly2Val	SP		0.055		
7	p.Ser5GlyfsX136	SP	rs797045079; (*1*)	1.000	0.4	Low
8	p.Ser5Leu	SP	rs1296229818	0.263	0.8	
9	p.Ser5Trp	SP		0.039		
10	p.Gly6Ala	SP	rs1267076673	0.079	0.7	
11	p.Gly6Arg	SP	rs2049627437	0.027	0.8	
12	p.Arg7Ser	SP	rs1285068027	0.001	0.7	
13	p.Arg7Gly	SP		0.001		
14	p.Arg7Leu	SP	rs1451926480	0.001	2.2	
15	p.Arg8GlyfsX134	SP	(*2*)	1.000	0.4	Low
16	p.Arg8Leu	SP	rs2049627704	0.001	1.5	
17	p.Arg8Trp	SP	rs1333116255	0.001	7.8	
18	p.Gly9Arg	SP	rs1320210312	0.004	0.8	
19	p.Gly9Trp	SP	rs1223694748	0.008		
20	p.Gly9Glu	SP		0.010	3.0	
21	p.Gly11Arg	SP	rs1405957884	0.000	0.4	
22	p.Leu13Pro	SP	rs1187548350	0.001	**96**[a]	
23	p.Leu13_Leu14del	SP	rs900084108; (*2*)	1.000	6.6	Low
24	p.Leu13_Leu16del	SP	rs751352152; (*3*)	1.000	0.8	Low
25	p.Leu14Pro	SP	rs1207951348	0.000	0.4	
26	p.Leu14_Leu22del	SP	rs90879686	1.000	1.6	
27	p.Pro15_16PL(2)indel	SP	rs522691783	1.000	**10**	
28	p.Pro15_Leu21del	SP	rs1245868974	1.000	0.8	
29	p.Pro15Ser	SP	rs1193133040	0.001	0.4	
30	p.Pro15Leu	SP	rs1355518990	0.000	1.4	
31	p.Pro15Gln	SP		0.000		
32	p.Leu16_Pro23indel	SP	rs983649759; (*2*)	1.000	**19**	Low
33	p.Leu16Pro	SP	rs1352305726	0.000	3.3	
34	p.Pro17Arg	SP	rs1441805434	0.084	0.9	
35	p.Pro17Ser	SP	rs1599136248	0.001	0.4	
36	p.Leu18_L20ins	SP	rs532691783; (*4*)	1.000	6.0	86 (*4*)
37	p.Leu19_Pro24ins	SP	rs1437482955	1.000	0.4	
38	p.Leu19Pro	SP	rs1157043147	**0.694**	**13**	
39	p.Leu20Serfs	SP	rs752411292	1.000	1.6	
40	p.Leu20Trp	SP	rs770640756	**0.624**	1.4	
41	p.Leu21Pro	SP	(*2*)	**0.797**	0.4	Low
42	p.Leu22Val	SP	rs2049629392	0.068	0.7	
43	p.Pro23Ser	SP	rs1288779128	0.020	0.0	
44	p.Pro23Ala	SP		0.001		
45	p.Pro23Leu	SP	rs2049629518	0.000	0.4	
46	p.Pro24Thr	SP	rs2049629589	0.006	**72**[a]	
47	p.Pro24del	SP	rs1440772953	1.000	1.6	
48	p.Gln25X	SP	rs1237545952	1.000	1.6	
49	p.Gln25Leu	SP	rs968327653	0.000	3.3	

50	p.Ala27Gly	SP	rs774092241	0.220	8.8	
51	p.Ala27Val	SP		0.002		
52	p.Ala27Thr	SP	rs2049629805	0.002	0.8	
	Combined frequency of damaging ACE mutations MAF				**50.8**	
**I. Combined frequency of damaging mutations, % in population**					**0.05%**	

#	Genetic position	Amino acid position (mature protein)	Polymorphism or (*reference*)	PolyPhen-2 Score (HVAR)	Minor Allele Frequency, per 100 000	Blood ACE, % of M	AD
**II. Indels or stop codons in mature ACE**							
1	p.Leu34Profs	L5Pfs	rs1459096726		0.4		
2	p.Gln51X	Q22X	rs1184203291		0.8		
3	p.Ser52Glnfs	S23Qfs	rs1189819056		0.8		
4	p.Gln59X	Q30X	rs868134438		0.8		
5	p.Gln63X	Q34X	rs1278390159		0.8		
6	p.Thr113Phefs	T84Ffs	rs1232118105		0.8		
7	p.Glu116X	Q87X	rs747960753		1.3		
8	p.Glu138X	Q109X	rs779422412		0.5		
9	p.Arg149Leufs*54	R120LfsX54	rs778759606; (*2,5*)	insTTAGC	4.2	Low	AD (*5*)
10	p.Tyr151del	Y122del	rs750908161		2.8		
11	p.Ser179Serfs	S150Sfs	rs1441192851		0.4		
12	p.Arg180X	R151X	rs779454500		0.8		
13	p.Ser181Thrfs	S152Tfs	rs56397551		0.4		
14	p.Trp189X	W160X	rs765401595		0.8		
15	p.Glu190Argfs	E161Rfs	rs1402956277		0.4		
16	p.Trp230X	W201X	rs757421466		1.6		
17	p.Tyr244Profs	Y215Pfs	rs1214574142		3.2		
18	p.Tyr251Phefs	Y222Ffs	rs1285331787		0.4		
19	p.Arg265X	R236X	rs138873311; (*2*)		1.2	Low	
20	p.Tyr266X	Y237X	rs121912704; (*6,7*)		0.8	Low (*6*)	
21	p.Asp268Glyfs	D239Gfs	rs1462640798		0.4		
22	p.Arg269ins	R240Yins	rs769080277		0.4		
23	p.Arg274GlyfsX117	R245Gfs	(*8*)		0.4	Low	
24	p.Met285Valfs	M256Vfs	rs769816155		0.4		
25	p.Phe300Serfs	F271Sfs	rs1387186484		0.4		
26	p.Glu315X	E286X	rs760913528		0.4		
27	p.Trp317X	W288X	rs1414333467		0.8		
28	p.Ala326Glufs	A297Efs	rs1474671878		1.6		
29	p.Glu328del	E299del	(*2*)		0.4	Low	
30	p.Met338Profs	M309Pfs	rs1196439789		0.4		
31	p.Trp343X	W314X	rs200225958; (*2,5*)		0.8	Low	AD (*5*)
32	p.Ser346GlufsX47	S317Efs	rs1331062614; (*2*)		0.4	Low	
33	p. Val358del	V329del	rs770842341		0.4		
34	p.Cys359Valfs	C330Vfs	rs774181017		0.4		
35	p.Gln396X	Q367X	rs76906391		0.4		
36	p.Ala412Glyfs	A383Gfs	rs759192800		0.8		
37	p.Glu432Profs	E403Pfs	rs1388420671		0.8		
38	p.Lys436X	K407X	rs1427700343		1.0		
39	p.Leu440ProfsX15	L411Pfs	rs387906576; (*6*)		0.4	Low	
40	p.Asp441fs	D412fs	(*5*)		0.4		AD (*5*)
41	p.Arg442Valfs	R413Vfs	rs1442562714		0.4		
42	p.Leu454X	L425X	rs1404415405		0.8		
43	p.Ile462Asnfs	I433Nfs	rs140992429		0.8		
44	p.Phe464Alafs	F435Afs	rs753436653		0.8		
45	p.Trp474X	W445X	rs762742726		0.8		
46	p.Val478Valfs	V449fs	rs1246593224		0.4		
47	p.Pro485Leufs	P456fs	(*9*)		0.4	Low	
48	p.Ser486Profs	S457Pfs	rs758933315		2.4		
49	p.Asp491Glufs	D462Efs	rs1257147672		0.4		
50	p.Trp492X	W463X	rs1446625916		5.6		
51	p.Arg496X	R467X	rs397514688; (*2*)		0.4	Low	

52	p.Gln500X	Q471X	rs2049747561		0.7		
53	p.Gln500Argfs	Q471Rfs	rs748348196		2.4		
54	p.Pro505Del	P576del	rs766909364		7.6		
55	p.Arg508X	R479X	rs367797185; (*2*)		3.2	Low	
56	c.1709+5G>T	Abn. splicing	(*2*)		0.4	Low	
57	p.Gln537X	Q508X	rs1460738029		0.4		
58	p.537_538QF(3)	Q508_F509ins_	rs771053807		0.8		
59	p.Glu539X	E510X	rs1474365721		0.4		
60	p.Glu547X	E518X	rs1319348585		0.4		
61	p.Trp581X	W552X	rs768467806		0.4		
62	p.Trp581Glyfs	W552Gfs	(*2*)		0.4	Low	
63	p.Gln597X	Q568X	rs2029862662		0.4		
64	p.Lys601AsnfsX40	K572Nfs	(*2,10*)		0.8	Low (*2*)	
65	p.Trp609X	W580X	rs769466912		0.4		
66	p.Glu613X	E584X	rs935167896		3.0		
67	p.Gln616X	Q587X	rs762769560		0.4		
68	p.Gly622Alafs	G593Afs	rs772014965		**26**		
69	p.Trp628X	W599X	rs745879536		0.4		
70	p.Glu655X	E526X	rs1371611657		0.8		
71	p.Trp664X	W635X	rs1419177611		0.4		
72	p.Trp672X	W643X	(*11*)			Low	
73	p.Gln692X	Q663X	rs1260448350		0.4		
74	p.Tyr700X	Y671X	rs780199864		2.0		
75	p.Ile717Glnfs	I688Qfs	rs1219522144; (*2*)		0.8	Low	
76	p.Ile721LysfsX60	I692Lfs	(*2*)		0.4	Low	
77	p.Gln735X	Q706X	rs933390771		0.8		
78	p.Leu744Cysfs	L715Cfs	rs745767649		1.6		
79	p.Val756Argfs	V727Rfs	rs772172179		0.4		
80	p.Leu784Tyrfs	L755Yfs	rs1476011360		0.8		
81	p.Trp787X	W758X	rs775934699		0.8		
82	p.Glu788Argfs	E759Rfs	rs1482032796		0.8		
83	p.Arg791X	R762X	(*2,10*)		0.4	Low (*2*)	
84	p.Gln800X	Q771X	rs1568043111		0.4		
85	p.Tyr805X	Y776X	rs761458810		0.4	49	
86	p.Asp820Metfs	D791Mfs	rs757544710		0.8		
87	p.Trp825X	W796X	rs2030184791		0.4		
88	p.Arg826Serfs	R797Sfs	rs1441728993		0.4		
89	p.Gln836X	Q807X	rs1405858837		1.2		
90	p.Gln843Argfs	Q814Rfs	rs956900465		0.4		
91	p.Gln867X	Q838X	rs1413766379		0.4		
92	p.Asn882Serfs	N853Sfs	rs146430617		0.4		
93	p.Tyr892X	Y863X	rs762809850		0.4		
94	p.Pro897fs	P868fs	(*12*)		0.4	Low	
95	p.Trp941X	W912X	rs2030363266		0.8		
96	p.Ser944Glnfs	S915Qfs	rs1221050110		0.4		
97	p.Lys948_Pro949del	K919_P920del	rs1292471704		0.4		
98	p.Glu954Glyfs	E925Gfs	rs78007237904		0.4		
99	p.Gln994X	Q965X	rs2030522022		0.7		
100	p.Pro1003_Ala1010del	P974_A981del	rs746977732		0.4		
101	p.Leu1024fs	L995fs	(*5*)		0.4	Low	AD (*5*)
102	p.Ser1027Tyrfs	S998Wfs	rs1170915014		3.2		
103	p.Leu1032fs	L1003fs	(*12*)		0.4	Low	
104	p.Glu1041_Ser1044del	Q1012del	rs768525377		0.8		
105	p.Asp1058Tyrfs	D1029Yfs	(*5*)		0.4	Low	AD (*5*)
106	p.Pro1064Serfs	P1035Sfs	rs747724861		1.6		

107	p.Trp1072X	W1053X	rs773163752		0.8		
108	p.Trp1074X	W1055X	rs1266895232		0.8		
109	p.Trp1091X	W1062X	rs1411245193		0.4		
110	p. Gln1098X	Q1069X	rs1568047250		0.4		
111	p.Val1104fs	P1075SPfs	rs1447215842		0.4		
112	p.Val1130Glndel	V1101Qfs	rs772585024		0.8		
113	p.Val1130Metfs	V1101Mfs	rs762468887		2.4		
114	p.Ser1131Profs	S1102Pfs	rs775933853		2.4		
115	p.Gln1137X	Q1108X	rs1341633213		0.4		
116	p.Gln1144X	Q1115X	rs1386775881		0.4		
117	p.Asp1156fs	D1127Rfs	rs1403340480		0.4		
118	p.Tyr1158X	Y1129X	rs1381445771		0.4		
119	p.Tyr1158fs	Y1129Ifs	rs1367062284		0.8		
120	p.Lys1161fs	K1132Qfs	rs34743858		0.4		
121	p.Gln1165X	Q1136X	(*2,10*)		0.4	Low (*2*)	
122	p.Lys1172_Met1183de	K1143_M1154del	(*1*)		0.4	Low	
123	c.3503+1G>A	Abn. splicing	(*2*)		0.4	Low	
124	c.3691+1G>A	Abn. splicing	(*13*)		4.4	1133	
125	p.Gly1174AlsfsX12	G1145Afs	rs754265941;(*2,8*)		**47**	Low	
126	p.Trp1179X	W1150X	rs1406482731		0.4		
127	p.Gln1184X	Q1155X	rs20307701130		0.4		
128	p.Glu1217X	E1188X	rs534095139		0.8		
129	p.Trp1226X	W1197X[b]	rs769710002; (*14*)		0.4	1300	
130	p.Ser1238Pfs	S1209Pfs	(*5*)		0.4	Low	AD (*5*)
131	p.1248_1249LD(3)	L1252_D1253ins	rs780845846		0.4		
132	p.Gln1253X	Q1224X[b]	rs1174820268; (*15*)		0.4	1200	
133	p.Leu1276Ser1277del	L1247_S1248del	rs1367232864		0.6		
134	p.Gln1296X	Q1267X	rs1162756119		0.4		
			Sum of D		186.2		
**II. Combined frequency of damaging mutations, % in population**					**0.19%**		

#	Genetic position	Amino acid position (mature protein)	Polymorphism or (*reference*)	PolyPhen-2 Score (HVAR)	Minor Allele Frequency, per 100 000	Blood ACE, % of M
**III. All missense mutations (including damaging)**						
1	p.Leu30Ser	L1S	rs1196105733	0.374	0.8	
2	p.Leu30Phe	L1F	rs1450600177	**0.855**	0.7	
3	p.Asp31Glu	D2E	rs1200169472	0.000	0.4	
4	p.Pro32Thr	P3T	rs1395554180	0.002	1.1	
5	p.Gly33Arg	G4R	rs1363496774	**0.890**	0.4	
6	p.Pro36Thr	P7T	rs761292178	**0.853**	0.8	
7	p.Pro36Leu	P7L	rs769008922	0.914	1.9	
8	p.Asn38Ser	N9S	rs1327600431	0.001	0.9	
9	p.Ala41Thr	A12T	rs926499615	0.016	0.9	
10	p.Asp42Asn	D13N	rs376354160	0.029	3.6	
11	p.Asp42Glu	D13E	rs1188476738	0.018	0.8	
12	p.Glu43Ala	E14A	rs1310330954	**0.889**	0.8	
13	p.Ala44Thr	A15T	rs765456530	0.045	0.4	
14	p.Gly45Arg	G16R	rs750712925	0.999	2.9	Low (*16*)
15	p.Gly45Glu	G16E	rs763151946	**0.470**	0.8	
16	p.Ala46Thr	A17T	rs1156835126	**0.879**	3.3	
17	p.Glu47Arg	Q18R	rs767353320	0.003	4.3	
18	p.Phe49Leu	F20L	rs752407759	**0.858**	6.4	
19	p.Ala50Thr	A21T	rs1412092470	0.020	0.8	
20	p.Ser52Asn	S23N	rs777523880	0.027	0.8	
21	p.Ser52Arg	S23R	rs368265670	0.316	**65**	
22	p.Ser52Gly	S23G	rs756108093	0.049	0,8	
23	p.Tyr53Cys	Y24C	rs991760634	0.950	0.4	
24	p.Asn54Ser	N25S	rs756763242	0.173	6.0	
25	p.Ser55Pro	S26P	rs2049631560	0.047	0.4	
26	p.Ser55Phe	S26F	rs886053219	0.261	0.8	
27	p.Ser56Asn	S27N	rs1854521219	**0.503**	0.4	
28	p.Ala57Thr	A28T	rs2049632676	0.191	0.8	
29	p.Leu61Gln	L32Q	rs74552589	0.046	0.4	
30	p.Phe62Ser	F33S	rs1345758653	0.011	0.8	
31	p.Gln63His	Q34H	rs780601919	**0.618**	0.8	
32	p.Ser64Gly	S35G	rs747292160	0.104	0.4	
33	p.Val65Met	V36M	rs776943620	0.061	2.2	
34	p.Ala66Thr	A37T	rs1450630173	0.004	1.1	
35	p.Ala67Gly	A38G	rs887280103	0.974	1.9	
36	p.Ser68Ile	S39I	rs1331734032	0.920	0.4	
37	p.Ser68Arg	S39R	rs1170287329	**0.446**	4.7	
38	p.His71Tyr	H42Y	rs773257897	0.000	0.8	
39	p.Asp72Tyr	D43Y	rs752559637	**0.692**	5.9	
40	p.Asn74Asp	N45D	rs1331766879	0.242	0.7	
41	p.Asn74Ser	N45S	rs1235548322	**0.489**	0.8	
42	p.Ile75Asn	I46N	rs2049633221	0.974	**30**	
43	p.Ile75Phe	I46F	rs375602836	0.944	1.5	
44	p.Thr76Ser	T47S	rs1465491488	0.102	0.7	
45	p.Ala77Thr	A48T	rs867626302	0.009	2.2	
46	p.Glu78Gln	E49Q	rs778615098	0.236	3.7	
47	p.Arg82Leu	R53L	rs997125723	0.002	0.4	
48	p.Gln83Lys	Q54K	rs1344103805	0.032	0.8	

49	p.Glu84Lys	E55K	rs1345164089	**0.758**	0.8	
50	p.Glu85Lys	E56K	rs150382846	0.173	4.1	
51	p.Ala87Ser	A58S	rs1440389747	0.022	0.4	
52	p.Leu88Val	L59V	rs762976911	0.077	0.4	
53	p.Leu89His	L60H	rs765857431	0.914	2.9	
54	p.Ser90Gly	S61G	rs751050925	0.016	4.1	
55	p.Ser90Asn	S61N	rs1221928144	0.002	0.8	
56	p.Gln91Glu	Q62E	rs1414717313	0.122	0.7	
57	p.Glu92Gly	E63G	rs767149889	**0.598**	1.1	
58	p.Phe93Val	F64V	rs122622664	0.917	0.4	
59	p.Ala94Val	A65V	rs753078890	0.024	4.1	
60	p.Ala96Pro	A67P	rs756407366	0.332	0.4	
61	p.Ala96Val	A67V	rs1193074438	0.003	0.4	
62	p.Trp97Ser	W68S	rs2049646473	0.086	0.4	
63	p.Trp97Cys	W68C	rs1471264963	**0.617**	0.4	
64	p.Gln99Glu	Q70E	rs749853877	0.002	5.7	
65	p.Lys100Glu	K71E	rs1407660027	0.079	0.4	
66	p.Ala101Thr	A72T	rs757757495	0.357	1.6	
67	p.Glu103Asp	E74D	rs1439594637	0.017	0.4	
68	p.Tyr105Cys	Y76C	rs1220739409	**0.769**	3.2	
69	p.Glu106Gly	E77G	rs1279075366	0.001	0.7	
70	p.Pro107Arg	P78R	rs772201818	0.383	2.9	
71	p.Glu110His	E81H	rs780364983	0.077	0.4	
72	p.Thr113Met	T84M	rs1271259475	0.174	0.4	
73	p.Asp114His	D85H	rs1568035450	0.987	0. 4	
74	p.Pro115Arg	P86R	rs1334067073	**0.520**	0.4	
75	p.Pro115Ser	P86S	rs1188044442	0.053	0.8	
76	p.Arg118Cys	R89C	rs1439803774	0.989	0.4	
77	p.Arg118Leu	R89L	rs773121528	0.376	1.6	
78	p.Ile120Phe	I91F	rs762839851	0.354	0.9	
79	p.Ile120Thr	I91T	rs770863321	0.203	0.4	
80	p.Ile121Val	I92V	rs539067889	0.050	2.6	
81	p.Ile121Thr	I92T	rs1416423148	0.922	0.4	
82	p.Gly122Arg	G93R	rs1170830801	0.030	4.7	
83	p.Ala123Asp	A94D	rs2049648175	0.012	0.4	
84	p.Val124Ala	V95A	rs2049648205	0.217	0.7	
85	p.Arg125Cys	R96C	rs759033270	**0.801**	3.4	
86	p.Arg125Pro	R96P	rs904130482	**0.667**	0.4	
87	p.Thr126Iso	T97I	rs936814960	0.048	0.5	
88	p.Gly128Ala	G99A	rs767085054	**0.833**	2.3	
89	p.Ala135Thr	A106T	rs752416873	0.018	1.0	
90	p.Lys136Glu	K107E	rs2049648986	0.109	0.8	
91	p.Lys136Thr	K107T	rs760310248	**0.634**	1.5	
92	p.Arg137Gly	R108G	rs764488884	**0.747**	4.0	
93	p.Arg137Trp	R108W		1.000		
94	p.Arg137Gln	R108Q	rs556087296	0.051	2.7	
95	p.Gln139Glu	Q110E	rs750481872	0.071	1.6	
96	p.Asn141Lys	N112K	rs746469812	**0.839**	3.0	
97	p.Ser145Ile	S116I	rs768306471	0.994	0.4	
98	p.Ser145Arg	S116R	rs776279706	0.938	0.9	
99	p.Asn146Ile	N117I	rs761366166	0.148	0.8	
100	p.Met147Val	M118V	rs201716509	0.916	0.8	
101	p.Met147Thr	M118T	rs773425152	0.996	0.8	
102	p.Met147Arg	M118R		1.000		
103	p.Ser148Asn	S119N	rs147057007	0.006	0.4	

104	p.Arg149Leu	R120L	rs766945182	0.146	6.4	
105	p.Ile150Met	I121M	rs1370591668	**0.870**	0.4	
106	p.Ser152Thr	S123T	rs75214560	0.258	0.4	
107	p.Thr153Ala	T124A	rs767705427	0.059	1.2	
108	p.Thr153Ile	T124I	rs201277497	**0.817**	1.2	
109	p.Ala154Thr	A125T	rs13306087	0.330	**84**	
110	p.Ala154Val	A125V	rs569318874	**0.486**	0.4	
111	p.Lys155Asn	K126N	rs143320537	**0.727**	**32**	
112	p.Val156Phe	V127F	rs745608171	0.940	0.4	
113	p.Cys157Ser	C128Ser	rs2049664994	**0.879**	1.5	
114	p.Asn160His	N131H	rs768220716	0.154	1.6	
115	p.Asn160Ser	N131S	rs117134739	0.032	3.8	
116	p.Thr162Ile	T133I	rs1459787773	0.296	1.6	
117	p.Ala163Asp	A134D	rs1378946788	0.045	1.1	
118	p.Trp166Arg	W137R	rs1015195326	0.911	0.8	
119	p.Ser167Phe	S138F	rs1362069107	0.924	0.7	
120	p.Leu168Pro	L139P	rs139076951	0.966	**32**	
121	p.Thr173Ile	T144I	rs1277204441	**0.521**	2.4	
122	p.Asn174Ser	N145S	rs751322397	0.004	3.0	
123	p.Ile175Asn	I146N	rs1346356853	**0.894**	2.1	
124	p.Leu176Pro	L147P	rs755647501	0.997	0.4	
125	p.Ser178Phe	S149F	rs1305248868	0.198	1.6	
126	p.Ser179Leu	S150L	rs374910265	0.993	0.8	
127	p.Arg180Gln	R151Q	rs369022610	0.299	4.4	
128	p.Ser181Thr	S152T	rs1213510652	0.032	0.8	
129	p.Tyr182Cys	Y153C	rs772645129	0.999	0.4	
130	p.Ala183Thr	A154T	rs12720754	0.230	183	
131	p.Met184Val	M155V	rs776669133	0.002	0.8	
132	p.Met184Iso	M155I	rs1172383595	0.002	0.7	
133	p.Leu185Phe	L155F	rs762060056	0.999	1.2	
134	p.Ala188Thr	A159T	rs1568036282	0.294	0.7	
135	p.Glu190Gln	E161Q	rs751371256	0.274	1.2	
136	p.His193Arg	H164R	rs1461565755	**0.853**	0.4	
137	p.Asn194Ser	N165S	rs2049677786	**0.504**	0.4	
138	p.Ala195Thr	A166T	rs767340249	0.129	2.8	
139	p.Ala195Val	A166V	rs376986357	0.101	8.3	
140	p.Ala196Val	A167V	rs756060281	0.006	0.8	
141	p.Gly197Asp	G168D	rs753361228	0.994	2.8	
142	p.Iso198Leu	I169L	rs778647989	0.002	0.4	
143	p.Pro199Leu	P170L	rs553520266	**0.834**	4.8	
144	p.Lys201Thr	K172T	rs769174358	0,997	0.8	
145	p.Lys201Glu	K172E	rs2049677865	0.978	0.4	
146	p.Pro202Leu	P173L	rs148460287	**0.785**	**32**	
147	p.Leu203Pro	L174P	rs1175840645	**0.556**	0.7	
148	p.Tyr204Asn	Y175N	rs2049678156	1.000	1.4	
149	p.Glu205Lys	E176K	rs763223753	0.038	3.6	
150	p.Asp206Val	D177V	rs767268916	0.177	0.4	
151	p.Ala209Thr	A180T	rs775328930	0.008	0.8	
152	p.Ser211Gly	S182G	rs760563261	0.412	0.8	
153	p.Ser211Ile	S182I	rs148144906	0.976	0.8	
154	p.Asn212Asp	N183D	rs764076582	0.988	2.0	
155	p.Asn212Lys	N183K	rs753408382	1.000	3.2	
156	p.Ala214Thr	A185T	rs142677199	0.962	3.2	
157	pAla214Gly	A185G	rs1354264426	**0.480**	1.6	
158	p.Lys216Arg	K187R	rs2049678808	0.006	6.0	

159	p.Gln217Glu	Q188E	rs1209300158	0.011	0.8		
160	p.Gly219Ser	G190S	rs750097881	0.994	2.1		
161	p.Gly219Val	G190V	rs769812428	0.998	0.4		
162	p.Phe220Leu	F191L	rs772619837	0.239	0.4		
163	p.Asp222Glu	D193E	rs751284054	**0.857**	0.4		
164	p.Asp222Asn	D193N	rs765803965	**0.812**	0.4		
165	p. Thr223Met	T194M	rs759167880	0.351	2.3		
166	p.Ala225Ser	A196S	rs753023714	0.383	5.2		
167	p.Tyr226His	Y197H	rs756638375	0.980	0.4		
168	p.Trp227Arg	W198R	rs111998398	1.000	0.4		
169	p.Arg228Cys	R199C	rs141543325; (*7*)	0.994	**24**		
170	p.Arg228His	R199H	rs1455284992	0.987	1.6		
171	p.Ser229Phe	S200F	rs1376819053	0.441	0.4		
172	p.Trp230Cys	W201C	rs757421466	**0.635**	0.8		
173	p.Asn232Thr	N203T	rs1160172583	0.012	0.4		
174	p.Ser233Thr	S204T	rs901989090	0.022	0.4		
175	p.Ser233Phe	S204F	rs998995786	0.959	0.4		
176	p.Pro234Ser	P205S	rs778841130	0.146	0.8		
177	p.Glu237Lys	E208K	rs773728684	0.050	1.6		
178	p.Asp239Tyr	D210Y	rs77294580rs	0.026	0.4		
179	p.Glu241Gln	E212Q	rs763411587	0.239	0.4		
180	p.His242Tyr	H213Y	rs749170321	0.004	0.4		
181	p.Tyr244Cys	Y215C	rs3730025; (*7,16-18*)	1.000	**1068**	73 (*4,19*)	AD (*17-18*)
182	p.Tyr244His	Y215H	rs2049686933	0.998	0.4		
183	p.Gln245Glu	Q216E	rs2049687041	0.004	1.1		
184	p.Leu247Pro	L218P	rs1469557705	0.997	0.4		
185	p.Glu248Gln	E219Q	rs775849960	0.144	0.8		
186	p.Pro249Leu	P220L	rs760966452	0.999	0.4		
187	p.Leu250Val	L221V	rs1409716305	0.979	0.8		
188	p.Leu250Pro	L221P	s764359224	1.000	0.4		
189	p.Tyr251His	Y222H	rs1279450935	1.000	0.4		
190	p.Leu252Pro	L223P	rs2049687508	0.997	0.4		
191	p.Val256Val	A227V	rs758714422	0.991	0.8		
192	p.Phe257Leu	F228L	rs780365048	0.415	0.4		
193	p.Val258Ile	V229I	rs747960808	**0.546**	1.9		
194	p.Arg259Cys	R230C	rs777408360	1.000	1.2		
195	p.Arg259His	R230H	rs370903033; (*2,9*)	0.995	1.2	Low	
196	p.Arg260Cys	R231C	rs147670020	**0.805**	0.8		
197	p.Arg260His	R231H	rs150011877	0.426	2.4		
198	p.Ala261Ser	A232S[c]	rs4303; (*20*)	**0.848**	112		
199	p.Arg261Val	A232V	rs564933233	**0.589**	0.8		
200	p.His263Tyr	H234Y	rs1478780828	0.010	1.1		
201	p.Arg264Cys	R235C	rs1249291422	0.972	2.8		
202	pArg264His	R235H	rs776828648	0.314	0.8		
203	p.Tyr266Cys	Y237C	rs373616533	1.000	0.8		
204	p.Gly267Arg	G238R	rs149412997; (*7*)	0.973	**33**		
205	p.Asp268Asn	D239N	rs1403582878	0.146	0.7		
206	p.Asp268Glu	D239E	rs766561924	0.025	0.4		
207	p.Tyr270Asn	Y241N	rs2049688901	0.119	6.0		
208	p.Ile271Val	I242V	rs530535736	0.065	0.8		
209	p.Gly275Arg	G246R	rs1316614976	**0.897**	2.1		
210	p.Pro276Ala	P247A	rs777657188	0.371	0.7		
211	p.Ile277Val	I248V	rs757233793	**0.753**	1.2		
212	p.Pro278Thr	P249T	rs1229110590	1.000	0.4		
213	p.His280Arg	H251R	rs1303022378	0.999	0.8		

214	p.Leu281Gln	L252Q	rs778759109	1.000	0.8		
215	p.Leu282Pro	L253P	rs1040577967	0.999	1.1		
216	p.Met285Val	M256V	rs923234679	**0.712**	0.4		
217	p.Met285Thr	M256T	rs747832023	1.000	1.2		
218	p.Met285Ile	M256I	rs1749235389	**0.712**	0.8		
219	p.Trp286Gly	W257G	rs1365148386	0.999	0.4		
220	p.Ala287Ser	A258S	rs1218416633	**0.690**	0.4		
221	p.Ala287Val	A258V	rs770340552	0.987	0.4		
222	p.Gln288Arg	Q259R	rs199591851; (*2,9*)	0.998	**74**	68 (*4*)	
223	p.Ser289Asn	S260N	rs763587114	0.354	0.4		
224	p.Trp290Ser	W261S	rs1266264733	1.000	0.4		
225	p.Glu291Lys	E262K	rs1464640594	0.194	0.4		
226	p.Glu291Ala	E262A	rs771517816	0.172	0.4		
227	p.Asp295Asn	D266N	rs989500910	0.308	0.4		
228	p.Met296Val	M267V	rs1190943736	0.011	0.4		
229	p.Val297Met	V268M	rs61740982	0.267	4.8		
230	p.Val298Met	V269M	rs752924819	0.054	1.1		
231	p.Pro299Thr	P270T	rs760860250	0.983	0.4		
232	p.Asp302Tyr	D273Y	rs139813210	**0.890**	0.4		
233	p.Pro304Leu	P275L	rs1419966263	0.105	0.4		
234	p.Asn305Ser	N276S	rs141186617	0.000	**11**		
235	p.Leu306Arg	L277R	rs1044992578	0.315	0.4		
236	p.Asp307Asn	D278N	rs532619151	**0.875**	0.4		
237	p.Asp307Gly	D278G	rs754511687	0.995	0.8		
238	p.Thr309Ala	T280A	rs747796419	**0.700**	0.4		
239	p.Ser310Gly	S281G	rs144137849	0.009	**18**		
240	p.Ser310Asn	S281N	rs774789681	0.003	0.4		
241	p.Thr311Ser	T282S	rs746377185	0.008	1.2		
242	p.Met312Val	M283V	rs367998749	**0.738**	2.4		
243	p.Met312Thr	M283T	rs201588518	0.999	1.6		
244	p.Gly316Cys	G287C	rs561813163	0.994	0.4		
245	p.Gly316Val	G287V	rs2049710108	0.998	0.4		
246	p.Ala319Ser	A290S	rs34126458	0.120	**90**		
247	p.Ala319Val	A290V	rs2049710282	0.019	0.4		
248	p.Thr320Ala	T291A	rs1384833654	0.030	0.4		
249	p.Thr320Met	T291M	rs139137100	0.163	0.7		
250	p.His321Tyr	H292Y	rs2049710489	0.102	0.7		
251	p.Met322Val	M293V	rs1391141938	**0.738**	0.8		
252	p.Arg324Trp	R295W	rs35141294	0.975	202		
253	p.Arg324Gln	R295Q	rs374029266	0.028	3.2		
254	p.Val325Met	V296M	rs771762179	0.207	0.4		
255	p.Ala326Thr	A297T	rs1273653682	0.940	1.6		
256	p.Glu328Lys	E299K	rs992848550	0.240	0.8		
257	p.Phe330Leu	F301L	rs763740829	0.954	3.6		
258	p.Thr331Ser	T302S	rs1444816395	0.030	0.7		
259	p.Leu333Val	L304V	rs983222441	**0.824**	0.7		
260	p.Leu333Gln	L304Q	rs761390621; (*2*)	1.000	0.7	Low	
261	p.Glu334Gln	E305Q	rs1336871330	0.295	0.7		
262	p.Pro337Ser	P308S	rs764882826	0.077	0.4		
263	p.Pro337Leu	P308L	rs750754792	**0.626**	1.2		
264	p.Met338Val	M309V	rs1414191617	0.307	1.5		
265	p.Met338Ile	M309I	rs1422578392	**0.797**	0.7		
266	p.Pro339Ser	P310S	rs1455859263	0.392	0.8		
267	p.Glu341Gln	E312Q	rs201456235	0.172	1.2		
268	p.Phe342Ser	F313S	rs755446607	1.000	0.4		

269	p.Ser346Leu	S317L	rs781272495	0.994	1.2	
270	p.Leu348Pro	L319P	rs769913687	0.995	0.4	
271	p.Glu349Ala	E320A	rs778126198	0.203	0.4	
272	p.Lys350Gln	K321Q	rs2049712159	0.060	0.4	
273	p.Pro351Leu	P322L	rs2229839; (*7*)	**0.832**	**24**	
274	p.Asp353Asn	D324N	rs148193919	0.100	**22**	
275	p.Gly354Arg	G325R	rs56394458; (*7*)	0.998	780	62 (*4*)
276	p.Arg355Trp	R326W	rs776297611	0.993	2.0	
277	p.Arg355Gln	R326Q	rs761322765	**0.487**	1.6	
278	p.Glu356Lys	E327K	rs553718986	0.014	0.4	
279	p.Val357Met	V328M	rs1273328602	0.296	0.7	
280	p.Val358Met	V329M	rs764651696	0.928	1.2	
281	p.His360Tyr	H331Y	rs199690936	0.442	0.4	
282	p.His360Arg	H331R	rs2049713014	**0.456**	0.4	
283	p.Ala361Thr	A332T	rs546455400	0.992	6.0	
284	p.Ser362Thr	S333T	rs1274888100	**0.460**	0.8	
285	p.Ser362Trp	S333W	rs142328237; (*21*)	1.000	6.8	71 (*21*)
286	p.Ala363Pro	A334P	rs1443267419	0.999	0.4	
287	p.Ala363Val	A334V	rs370491569	0.999	1.2	
288	p.Thr364Cys	W335C	rs1325867826	0.996	0.4	
289	p.Asn368Ser	N339S	rs369713789	0.098	0.8	
290	p.Asn368Lys	N339K	rs1482676646	**0.557**	1.1	
291	p.Arg369Gly	R340G	rs373357172	0.098	1.5	
292	p.Lys370Arg	K341R	rs1176719016	0.008	0.4	
293	p.Arg373Ser	R344S	rs189243320	1.000	3.2	
294	p.Lys375Arg	K345R	rs778168348	0.983	0.4	
295	p.Arg379Trp	R350W	rs750724647	0.983	4.4	
296	p.Arg379Gln	R350Q	rs13306085	0.983	2.4	
297	p.Val380Asp	V351D	rs752385390	**0.776**	0.4	
298	p.Thr381Met	T352M	rs150466411; (*7*)	0.998	**85**	
299	p.Met382Val	M353V	rs370890237	0.236	0.4	
300	p.Met382Thr	M353T	rs2049726728	0.291	0.4	
301	p.Met382Ile	M353I	rs770475936	0.316	0.4	
302	p.Asp383Tyr	D354Y	rs2049726795	0.947	1.1	
303	p.Asp383Gly	D354G	rs374899854	0.215	0.4	
304	p.Thr387Ala	T358A	rs1347736201	0.355	1.6	
305	p.His389Gln	H360Q	rs1405848294	1.000	0.7	
306	p.Met392Thr	M363T	rs138418851	0.991	4.0	
307	p.Gly393Asp	G364D	rs2049727156	0.999	0.7	
308	p.His394Arg	H365R	rs775932125	0.977	0.8	
309	p.Ile395Met	I366M	rs760885341	**0.896**	0.4	
310	p.Gln400Lys	Q371K	rs771386010	0.334	1.2	
311	p.Gln400Arg	Q371R	rs548450663	0.436	0.4	
312	p.Tyr401Cys	Y372C	rs765449601	0.986	1.2	
313	p.Lys402Met	K373M	rs1329993082	0.940	0.8	
314	p.Asp403Ala	D374A	rs763292265	0.045	0.8	
315	p.Leu404Val	L375V	rs1229622121	0.112	0.7	
316	p.Pro405Arg	P376R	rs766454164	0.996	1.5	
317	p.Val406Ile	V377I	rs201117983	0.050	**31**	
318	p.Ser407Phe	S378F	rs570424963	0.354	1.5	
319	p.Leu408Pro	L379P	rs2049728056	0.999	0.4	
320	p.Arg409Cys	R380C	rs199746395	1.000	2.4	
321	p.Arg409His	R380H	rs371833006	0.995	4.8	
322	p.Arg410Trp	R381W	rs370836540	**0.783**	2.8	
323	p.Arg410Gln	R381Q	rs145172277	0.004	**75**	

324	p.Gly411Arg	G382R	rs1347844823	1.000	0.4	
325	p.Ala412Ser	A383S	rs779643154	0.987	0.8	
326	p.Asn413Ser	N384S	rs1308632106	**0.848**	**15**	
327	p.Asn413Lys	N384K	rs1352223425	1.000	1.6	
328	p.Gly415Ser	G386S	rs149252911	0.990	5.6	
329	p.Gly415Asp	G386D	rs1350271986	0.999	0.4	
330	p.His417Arg	H388R	rs1229041283	0.910	0.4	
331	p.Glu418Lys	E389K	rs1599141410	0.998	**55**	Korean
332	p.Ala419Ser	A390S	rs1311367540	0.963	1.1	
333	p.Ile420Thr	I391T	rs144494842	0.996	**22**	
334	p.Ile420Val	I391V	rs2049728955	0.266	0.4	
335	p.Gly421Glu	G392E	rs2049729061	0.999	6.0	
336	p.Val423Met	V394M	rs148018765	**0.697**	7.6	
337	p.Ala425Val	A396V	rs372626836	**0.811**	2.8	
338	p.Leu426Arg	L397R	rs1295075641	1.000	0.8	
339	p.Ser427Leu	S398L	rs1484293906	0.995	2.4	
340	p.Val428Leu	V399L	rs1368163348	0.916	0.4	
341	p.Val428Gly	V399G	rs774484341	0.997	2.8	
342	p.Ser429Phe	S400F	rs1430341434	0.316	8.7	
343	p.Pro431Leu	P402L	rs2049729701	**0.907**	0.4	
344	p.His433Arg	H404R	rs763905584	0.928	0.8	
345	p.Leu434Val	L405V	rs753578845	**0.859**	**11**	
346	p.His435Leu	H406L	rs757195769	0.019	0.4	
347	p.Lys436Arg	K407R	rs765088731	0.077	0.4	
348	p.Gly438Ser	G409S	rs1051245483	**0.844**	0.8	
349	p.Gly438Asp	G409D	rs757908100	0.927	0.8	
350	p.Leu440Val	L411V	rs199697957	0.364	**12**	
351	p.Leu440Gln	L411Q	rs748465912	0.991	1.1	
352	p.Asp441Asn	D412N	rs770430455	0.045	0.8	
353	p.Arg442Cys	R413C	rs749779360	0.002	2.0	
354	p.Arg442His	R413H	rs35865660	0.001	132	
355	p.Asn445Asp	N416D	rs776411660	0.012	0.4	
356	p.Asn445Lys	N416K	rs2037090472	0.143	0.4	
357	p.Asp446Asn	D417N	rs2049730943	0.170	0.7	
358	p.Thr447Ala	T418A	rs761659396	0.004	1.5	
359	p.Thr447Met	T418M	rs746314800	0.046	4.8	
360	p.Ser449Gly	S420G	rs2049737716	0.104	0.4	
361	p.Ser449Thr	S420T	rs373076770	0.059	0.7	
362	p.Asp450Asn	D421N	rs185115105	0.310	0.8	
363	p.Ile451Val	I422V	rs1401663578	0.305	0.4	
364	p.Ile451Thr	I422T	rs1158360384	**0.820**	1.4	
365	p.Lys456Gln	K427Q	rs2049738166	0.121	6.0	
366	p.Ala458Val	A429V	rs1388880245	0.963	0.4	
367	p.Arg459Gln	R430Q	(*22*)			Low (*22*)
368	p.Ile462Phe	I433F	rs1370566904	**0.861**	0.8	
369	p.Ile462Asn	I433N	rs1289633744	1.000	0.4	
370	p.Phe464Ile	F435I	rs2049738685	**0.869**	0.4	
371	p.Gly468Cys	G439C	rs1170017440	0.996	1.4	
372	p.Val471Met	V442M	rs768818130	0.323	0.8	
373	p.Trp474Arg	W445R	rs772897915	0.999	0.4	
374	p.Arg475Cys	R446C	rs770628079	1.000	1.6	
375	p.Arg475His	R446H	rs774394975	1.000	4.3	
376	p.Gly477Glu	G448E	rs986987823	0.279	1.1	
377	p.Phe479Val	F450V	rs760413658	0.999	0.4	
378	p.Ser480Asn	S451N	rs886053220	0.055	0.4	

379	p.Arg482Cys	R453C	rs201540553; (*7*)	**0.649**	**19**	Low (*16*)
380	p.Arg482His	R453H	rs757694144	0.006	4.4	
381	p.Thr483Ile	T454I	rs2049740100	**0.833**	0.4	
382	p.Pro484Arg	P455R	rs1365864797	**0.615**	0.4	
383	p.Pro485Ala	P456A	rs202178737	0.059	9.1	
384	p.Pro485Arg	P456R	rs28730839; (*7*)	0.301	**48**	98 (*4*)
385	p.Pro485Leu	P456L	(*9*)			Low (*9*)
386	p.Ser486Phe	S457F	rs748305912	0.923	0.4	
387	p.Arg487Cys	R458C	rs149784122	0.972	**25**	
388	p.Arg487His	R458H	rs376430907	0.235	7.2	
389	p.Tyr488Ser	Y459S	rs948392443	0.999	1.1	
390	p.Asn489Asp	N460D	rs745820101	0.999	2.1	
391	p.Asn489Lys	N460K	rs145755731	1.000	0.8	
392	p.Asp491Asn	D462N	rs371335496	0.152	0.4	
393	p.Asp491Glu	D462E	rs1254289491	0.054	0.7	
394	p.Trp493Arg	W464R	rs2049741339	0.999	0.4	
395	p.Trp493Cys	W464C	rs1182135727	0.992	0.4	
396	p.Tyr494Asp	Y465D	rs760325775; (*23*)	0.011	2.4	700 (*23*)
397	p.Leu495Phe	L466F	rs374169715	0.974	0.4	
398	p.Arg496Gln	R467Q	rs761345398; (*22,24*)	1.000	1.9	Low (*22*)
399	p.Thr497Ala	T468A	rs766717973	0.382	0.4	
400	p.Lys498Arg	K469R	rs752110462	0.217	2.4	
401	p.Tyr499Cys	Y470C	rs779110765	0.999	2.8	
402	p.Gln500Arg	Q471R	rs1330033201	0.956	0.8	
403	p.Gly501Arg	G472R	rs886053221	1.000	5.0	
404	p.Gly501Glu	G472E	rs767656727	1.000	0.4	
405	p.Cys503Ser	C474S	rs1485835785	0.133	0.4	
406	p.Pro504Ser	P475S	rs778204413	0.912	1.6	
407	p.Pro504Leu	P475L	rs557514021	1.000	**20**	
408	p.Pro505Ala	P476A	rs148943954; (*7*)	0.939	**59**	147 (*4*)
409	p.Val506Ile	V476I	rs747001287	**0.480**	0.8	
410	p.Thr507Ala	T478A	rs1385293426	0.001	0.7	
411	p.Thr507Ser	T478S	rs1225189746	0.004	0.7	
412	p.Arg508Gln	R479Q	rs746397573	0.999	2.0	
413	p.Asn509Asp	N480D	rs769290119	0.071	0.4	
414	p.Glu510Lys	E481K	rs371544905	**0.847**	2.4	
415	p.Thr511Ala	T482A	rs762574298	0.027	1.6	
416	p.Asp514Asn	D485N	rs201762720	1.000	4.4	
417	p.Ala515Ser	A486S	rs144294634	0.978	**30**	
418	p.His520Asn	H491N	rs767844081	0.983	2.8	
419	p.His520Arg	H491R	rs1222739179	0.963	0.7	
420	p.Val521Ile	V492I	rs2049749257	0.072	0.4	
421	p.Pro522Leu	P493L	rs2049749316	0.994	6.0	
422	p.Asn523His	N494H	rs1053930450	**0.705**	0.8	
423	p.Asn523Ser	N494S	rs1195088899	0.121	0.7	
424	p.Val524Met	V495M	rs1198635867	**0.836**	1.4	
425	p.Val524Ala	V495A	rs12720746	0.150	4.0	
426	p.Thr525Ile	T496I	rs1251832995	**0.485**	0.4	
427	p.Thr525Ala	T496A	rs764244232	0.090	0.4	
428	p.Pro526Thr	P497S	rs754150700	0.997	0.7	
429	p.Tyr527Cys	Y498C	rs376323371	0.997	2.0	
430	p.Ile528Met	I499M	rs2029861083	**0.898**	0.4	
431	p.Arg529Ser	R500S	rs368074905	0.918	1.5	
432	p.Tyr530Cys	Y501C	rs745506888	0.999	1.2	
433	p.Tyr530His	Y501H	rs2029861365	0.942	0.4	

434	p.Phe531Cys	F502C	rs551801825	1.000	0.4	
435	p.Val532Leu	V503L	rs2029861375	**0.456**	0.7	
436	p.Phe534Leu	F505L	rs1390757637	0.274	0.7	
437	p.Val535Ile	V506I	rs1190471425	0.097	2.1	
438	p.Gln537Arg	Q508R	rs762937072	0.919	2.4	
439	p.Gln537His	Q508H	rs868856670	0.995	0.7	
440	p.Phe538Leu	F509L	rs769230286	0.963	0.4	
441	p.Gln539Lys	Q510K	rs1474365321	1.000	0.8	
442	p.His541Arg	H512R	rs776858777	0.998	0.4	
443	p.Glu542Gly	E513G	rs1453609198	**0.665**	0.7	
444	p.Ala543Val	A514V	rs765347178	**0.751**	0.8	
445	p.Ala543Ser	A514S	rs762055246	**0.547**	2.3	
446	p.Gly549Asp	G520D	rs1328713530	0.965	0.8	
447	p.Tyr550Cys	Y521C	rs753761783	**0.751**	1.2	
448	p.Gly552Ser	G523S	rs1339063327	**0.678**	0.7	
449	p.Gly552Asp	G523D	rs145152527	0.245	2.4	
450	p.Leu554Pro	L525P	rs2029861451	0.992	0.8	
451	p.His555Tyr	H526Y	rs778451287	**0.721**	1.2	
452	p.Cys557Arg	C528R	rs1012505443	1.000	1.9	
453	p.Asp558Asn	D529N	rs2029861470	**0.878**	0.4	
454	p.Ile559Val	I530V	rs1205538057	0.224	0.4	
455	p.Ile559Thr	I530T	rs2029861475	0.984	6.0	
456	p.Tyr560Cys	Y531C	rs745536540	0.996	0.4	
457	p.Arg561Trp	R532W	rs4314; (*7,20,25*)	**0.783**	**78**	500 (*25*)
458	p.Arg561Leu	R532L	rs780299861	0.082	1.2	
459	p.Ser562Pro	S533P	rs1599142834	0.993	0.4	
460	p.Thr563Pro	T534P	rs747313119	0.047	0.4	
461	p.Thr563Ile	T534I	rs769142434	0.179	**16**	
462	p.Lys564Glu	K535E	rs1171059871	0.002	0.7	
463	p.Lys564Thr	K535T	rs1599142842	0.025	**34**	Korean
464	p.Ala565Thr	A536T	rs777339023; (*26*)	0.976	4.8	
465	p.Ala565Val	A536V	rs2029861504	0.945	0.4	
466	p.Gly566Arg	G537R	rs748643856	1.000	0.8	
467	p.Gly566Glu	G537E	rs769805183	1.000	2.4	
468	p.Ala567Val	A538V	rs1422455629	0.358	**20**	
469	p.Ala567Thr	A538T	rs1399318948	0.059	0.4	
470	p.Leu569Val	L540V	rs1318295451	0.952	0.4	
471	p.Leu569Pro	L540P	rs773305413	1.000	0.4	
472	p.Arg570Trp	R541W	rs567828872	0.983	**15**	
473	p.Arg570Gln	R541Q	rs371599063	0.137	2.4	
474	p.Lys571Thr	K542T	rs2029862390	0.040	0.7	
475	p.Lys571Asn	K542N	rs777717910	0.096	3.6	
476	p.Leu573Pro	L544P	rs1365063879	0.999	0.8	
477	p.Gln574Lys	Q545K	rs2029862424	0.003	0.4	
478	p.Gln574Leu	Q545L	rs2029862431	0.046	0.7	
479	p.Gly576Ala	G547A	rs1243492273	0.989	0.4	
480	p.Ser577Pro	S548P	rs749450863	0.146	1.1	
481	p.Ser578Phe	S549F	rs2029862464	1.000	0.7	
482	p.Pro580Leu	P551L	rs897870088	**0.805**	0.8	
483	p.Pro580Thr	P551T	rs759719543	0.119	2.4	
484	p.Trp581Cys	W552C	rs1337718181	1.000	0.4	
485	p.Glu583Asp	E554D	rs1198303493	0.001	0.4	
486	p.Val584Met	V555M	rs2029862514	0.985	0.4	
487	p.Leu585Pro	L556P	rs776358299	0.998	0.8	
488	p.Lys586Glu	K557E	rs371414386	0.013	1.1	

489	p.Lys586Arg	K557R	rs1489606366	0.009	0.4	
490	p.Asp587Asn	D558N	rs1182072433	0.001	0.8	
491	p.Met588Val	M559V	rs1429588559	0.020	1.1	
492	p.Met588Thr	M559T	rs1171932485	0.092	0.4	
493	p.Gly590Ser	G561S	rs762585402	**0.898**	**34**	
494	p.Gly590Asp	G561D	rs1176792351	**0.882**	0.4	
495	p.Asp592Gly	D563G	rs12709426; (*27,28*)	0.047	382	
496	p.Asp592Asn	D563N	rs1450198005	0.022	0.4	
497	p.Leu594Pro	L565P	rs781708329	0.998	1.5	
498	p.Asp595Tyr	D566Y	rs753055168	0.992	0.4	
499	p.Ala596Ser	A567S	rs530248886	**0.562**	0.8	
500	p.Ala596Val	A567V	rs546796175	0.438	1.6	
501	p.Pro598Ser	P569S	rs988156346	**0.522**	0.4	
502	p.Pro598Leu	P569L	rs759009903	0.997	1.6	
503	p.Lys601Glu	K572E	rs1188841988	0.011	0.4	
504	p.Lys601Arg	K572R	rs776418026	0.037	0.8	
505	p.Phe603Ile	F574I	rs1178062715	0.999	0.4	
506	p.Thr607Asn	T578N	rs1477242406	0.085	0.4	
507	p.Gln608Pro	Q579P	rs1427973166	**0.771**	1.1	
508	p.Trp609Arg	W580R	rs1430977899	0.999	0.4	
509	p.Glu612Ala	E583A	rs773255356	0.991	2.0	
510	p.Asn614Ser	N585S	rs1568039509	**0.837**	0.4	
511	p.Asn617His	N588H	rs1455120932	0.209	0.4	
512	p.Asn617Ser	N588S	rs372497513	0.059	0.4	
513	p.Gly618Ser	G589S	rs111269527	0.435	2.5	
514	p.Glu619Lys	E590K	rs375452338	0.924	1.6	
515	p.Glu619Ala	E590A	rs1221968598	**0.696**	0.4	
516	p.Trp623Arg	W594R	(*2,10*)	1.000	0.4	Low (*2*)
517	p.Pro624Arg	P595R	rs972271442	0.246	1.1	
518	p.Glu625Lys	E596K	rs754396876	**0.680**	0.8	
519	p.Y626Asp	Y597D	rs757708886	0.114	2.6	
520	p.Tyr626Ser	Y597S	rs778975417	0.049	4.0	
521	p.Trp628Cys	W599C	rs758471657	0.994	1.1	
522	p.His629Pro	H600P	rs201594771; (*7*)	0.001	506	
523	p.His629Tyr	H600Y	rs2029862989	0.043	0.4	
524	p.Pro630Leu	P601L	rs142818229; (*4*)	0.988	4.1	154 (*4*)
525	p.Pro631Leu	P602L	rs749271989	0.018	3.4	
526	p.Asp634Glu	D605E	rs2029863038	0.000	6.0	
527	p.Asn635Ser	N606S	rs774004648	0.046	1.5	
528	p.Pro637Thr	P608T	rs759173310	0.998	0.4	
529	p.Pro637Leu	P608L	rs767112824	0.999	4.5	
530	p.Glu638Lys	E609K	rs760201372	0.125	9.8	
531	p.Gly639Val	G610V	rs754090770	0.410	0.8	
532	p.Gly639Ser	G610S	rs72845024; (*4*)	0.007	6.1	142 (*4*)
533	p.Ile640Arg	I611R	rs1307478617	**0.724**	0.4	
534	p.Asp641Val	D612V	rs759386648	0.012	0.4	
535	p.Leu642Val	L613V	rs2029871862	0.396	0.7	
536	p.Val643Met	V614M	rs767279985	**0.449**	0.7	
537	p.Val643Ala	V614A	rs1317871269	0.098	0.4	
538	p.Thr644Ser	T615S	rs752660066	0.001	1.6	
539	p.Asp645Asn	D616N	rs763603427	0.995	1.4	
540	p.Asp645Ala	D616A	rs1281544974	0.995	1.1	
541	p.Ala649Val	A620V	rs2029872327	0.998	0.7	

542	p.Phe652Tyr	F623Y	rs1393713094	**0.892**	0.7	
543	p.Val653Met	V624M	rs1248095456	0.058	0.4	
544	p.Glu654Lys	E625K	rs2029872533	**0.565**	0.4	
545	p.Glu655Asp	E626D	rs1487276305	**0.539**	0.4	
546	p.Glu655Lys	E626K	rs1371611657	**0.824**	0.7	
547	p.Tyr656His	Y627H	rs2029872680	0.994	6.0	
548	p.Arg658Trp	R629W	rs778684365	**0.581**	0.8	
549	p.Ser660Cys	S631C	rs147429960; (*7*)	0.242	**93**	
550	p.Ser660Ala	S631A	rs2029873057	0.000	0.4	
551	p.Gln661Leu	Q632L	rs1406304639	0.001	0.4	
552	p.Gln661His	Q632H	rs2029873234	0.004	0.4	
553	p.Val662Gly	V633G	rs1379553980	0.002	0.4	
554	p.Val663Leu	V634L	rs1178170347	0.002	0.4	
555	p.Glu666Lys	E637K	rs201804955	0.313	**33**	
556	p.Glu666Asp	E637D	rs2029873572	0.160	0.4	
557	p.Tyr667Cys	Y638C	rs1434646780	0.938	0.4	
558	p.Glu669Lys	E640K	rs769228405	**0.813**	**19**	
559	p.Glu669Gly	E640G	rs1266247312	0.921	0.7	
560	p.Ala670Asp	A641D	rs1177823963	0.075	0.4	
561	p.Asn671Thr	N642T	rs538715770	0.121	1.5	
562	p.Asn673Lys	N644K	rs564928656	0.005	0.4	
563	p.Asn675His	N646H	rs1245129210	0.076	0.4	
564	p.Ile678Val	I649V	rs371131106	**0.628**	8.4	
565	p.Thr679Ile	T650I	rs532375661	**0.635**	0.4	
566	p.Thr679Ala	T650A	rs771872424	0.023	0.8	
567	p.Glu681Asp	E652D	rs764154741	0.002	0.4	
568	p.Thr682Asn	T653N	rs753705010	0.000	0.8	
569	p.Lys684Asn	K655N	rs1453772021	0.003	0.4	
570	p.Met691Val	M662V	rs775191459	0.000	0.4	
571	p.Gln692Lys	Q663K	rs1260448350	0.015	0.7	
572	p.Ile693Val	I664V	rs2029953456	0.000	0.8	
573	p.Ala694Thr	A665T	rs764410917	0.327	0.8	
574	p.Asn695Ser	N666S	rs762256846	0.053	1.4	
575	p.His696Asn	H667N	rs1187097777	**0.688**	0.4	
576	p.His696Leu	H667L	rs2029954191	0.269	4.7	
577	p.Thr697Asn	T668N	rs765315607	0.928	1.1	
578	p.Gly701Ser	G672S	rs1172339137	0.999	3.2	
579	p.Thr702Asn	T673N	rs2029955964	0.001	0.4	
580	p.Gln703His	Q674H	rs751787326	0.089	2.6	
581	p.Ala704Val	A675V	rs756018163	**0.663**	0.8	
582	p.Arg705Gly	R676G	rs2029956646	0.006	0.0	
583	p.Arg705Lys	R676K	rs2029956870	0.003	0.4	
584	p.Lys706Arg	K677R	rs777673950	0.000	0.4	
585	p.Asp708Asn	D679N	rs1303374381	0.990	0.4	
586	p.Asn710Ser	N681S	rs770923059	0.000	1.7	
587	p.Gln711His	Q682H	rs2029958548	0.000	0.4	
588	p.Asn714Lys	N685K	rs778987310	0.012	0.8	
589	p.Thr716Ala	T687A	rs745422986	0.012	2.4	
590	p.Ile717Met	I688M	rs771585066	0.001	0.8	
591	p.Lys718Arg	K689R	rs1316503803	0.286	0.7	
592	p.Arg719Trp	R690W	rs200649158	1.000	5.6	
593	p.Arg719Gln	R690Q	rs371010069; (*7*)	0.995	2.4	

594	p.Ile721Val	I692V	rs769028657	0.006	0.4	
595	p.Ile721Met	I692M	rs1424703433	0.023	0.7	
596	p.Lys722Asn	K693N	rs188993222	0.123	0.8	
597	p.Gln725Glu	Q696E	rs139263584	0.393	1.2	
598	p.Gln725His	Q696H	rs2029962852	**0.904**	0.8	
599	p.Asp726Val	D697V	rs1420953232	0.946	0.4	
600	p.Leu727Arg	L698R	rs2029963533	**0.696**	6.0	
601	p.Arg729Trp	R700W	rs375232467	1.000	1.6	
602	p.Arg729Gln	R700Q	rs201527082	0.994	2.4	
603	p.Ala730Glu	A701E	rs767880620	0.999	8.4	
604	p.Ala730Ser	A701S	rs2029964544	0.968	6.0	
605	p.Ala731Val	A702V	rs1374995262	0.013	7.1	
606	p.Ala734Ser	A705S	rs199785479	0.011	0.8	
607	p.Leu737Gln	L708Q	rs757100327	0.999	0.4	
608	p.Glu738Val	E709V	rs1334538300	0.048	0.7	
609	p.Asn741Lys	N712K	rs779433192	0.996	0.4	
610	p.Lys742Glu	K713E	rs2029992650	0.001	0.7	
611	p.Lys742Arg	K713R	rs2029992890	0.001	0.7	
612	p.Ile743Met	I714M	rs1401450584	**0.846**	0.4	
613	p.Met747Thr	M718T	rs2029994732	0.997	6.0	
614	p.Glu748Lys	E719K	rs921762904	0.999	0.4	
615	p.Glu748Gly	E719G	rs1362206431	1.000	0.7	
616	p.Thr749Pro	T720P	rs1599146760	0.911	1.4	
617	p.Thr750Ile	T721I	rs2029996149	0.005	0.7	
618	p.Ser752Ile	S723I	rs2029996374	0.929	0.4	
619	p.Val753Met	V724M	rs140129129	0.075	4.3	
620	p.Ala754Pro	A725P	rs1202344569	0.943	0.7	
621	p.Ala754Val	A725V	rs1319509042	**0.830**	1.4	
622	p.Thr755Ile	T726I	rs200503880	0.095	0.4	
623	p.Thr755Ala	T726A	rs2029998101	0.002	5.0	
624	p.Val756Leu	V727L	rs773578992	0.364	0.4	
625	p.Val756Ala	V727A	rs377567489	**0.907**	0.7	
626	p.Cys757Tyr	C728Y	rs1232177858	0.999	0.8	
627	p.Pro759Ser	P730S	rs1180603936	0.000	0.7	
628	p.Pro759Gln	P730Q	rs143843660	0.001	0.8	
629	p.Cys763Tyr	C734Y	rs370481039	0.999	3.6	
630	p.Leu764Gln	L735Q	rs145819052; (*7*)	**0.662**	**25**	
631	p.Glu767Lys	E738K	rs148995315; (*7,28*)	**0.818**	**26**	
632	p.Glu767Gly	E738G	rs1421152152	**0.895**	1.4	
633	p.Asp769Gly	D740G	rs559834728	**0.613**	2.4	
634	p.Leu770Val	L741V	rs374146846	0.994	1.6	
635	p.Thr771Met	T742M	rs780755664	0.918	0.8	
636	p.Val773Met	V744M	rs143830698	0.018	8.4	
637	p.Met774Val	M745V	rs559585445	0.408	3.2	
638	p.Thr776Met	T747M	rs769940023	**0.796**	1.1	
639	p.Thr776Ala	T747A	rs199869667	0.043	2.8	
640	p.Arg778Trp	R749W	rs745724462	0.988	2.6	
641	p.Arg778Gln	R749Q	rs771819046	0.194	2.0	
642	p.Asp782Glu	D753E	rs760477392	0.000	1.2	
643	p.Leu784Ser	L755S	rs1162307952	0.228	0.4	
644	p.Trp785Gly	W756G	rs763670346	**0.666**	0.8	
645	p.Glu788Lys	E759K	rs761401927	0.002	9.2	

646	p.Glu788Asp	E759D	rs565463716	0.001	1.6	
647	p.Gly789Asp	G760D	rs953051570	**0.618**	0.4	
648	p.Arg791Gln	R762Q	rs755385604	0.996	2.0	
649	p.Asp792His	D763H	rs1291650441	**0.868**	0.8	
650	p.Lys793Met	K764M	rs748246753	0.324	0.8	
651	p.Ala794Thr	A765T	rs756178155	0.006	0.4	
652	p.Ala794Val	A765V	rs373970727	0.003	2.8	
653	p.Gly795Arg	G766R	rs2030160089	1.000	0.4	
654	p.Arg796Lys	R767K	rs2030160538	0.002	**12**	
655	p.Ala797Thr	A768T	rs1486364002	0.001	0.4	
656	p.Ala797Val	A768V	rs1455404812	0.003	0.4	
657	p.Iso798Val	I769V	rs117647476; (*7*)	0.004	213	
658	p.Leu799Phe	L770F	rs2030162108	0.939	0.4	
659	p.Leu799Pro	L770P	rs2030162361	0.939	1.1	
660	p.Gln800His	Q771H	rs567706604	0.003	1.2	
661	p.Pro803Leu	P774L	rs367822781	0.913	6.4	
662	p.Val806Met	V777M	rs769397961	1.000	4.2	
663	p.Leu808Phe	L779F	rs773031583	0.087	1.4	
664	p.Ile809Val	I780V	rs762647568	0.007	3.6	
665	p.Asn810Ser	N781S	rs1206246426	0.995	**22**	
666	p.Gln811Arg	Q782R	rs1255043434	0.017	0.7	
667	p.Ala812Val	A783V	rs751806358	**0.533**	0.4	
668	p.Arg814Trp	R785W	rs142799747	0.007	1.2	
669	p.Arg814Gln	R785Q	rs375979946	0.001	**11**	
670	p.Leu815Phe	L786F	rs935705219	0.967	0.7	
671	p.Asn816Ser	N787S	rs777776998	**0.853**	1.2	
672	p.Tyr818Phe	Y789F	rs369245002	0.015	2.0	
673	p.Val819Ala	V790A	rs1568043314	0.002	0.8	
674	p.Asp820Asn	D791N	rs1018632632	**0.784**	1.2	
675	pAla821Val	A792V	rs781086412	0.232	0.4	
676	p.Ala821Ser	A792S	rs2030182044	0.246	0.8	
677	p.Gly822Glu	G793E	rs1340223445	0.986	1.1	
678	p.Asp823Asn	D794N	rs995556379	**0.609**	0.4	
679	p.Asp823Val	D794V	rs777851729	0.032	2.8	
680	p.Ser824Pro	S795P	rs2030184043	**0.870**	0.4	
681	p.Ser827Cys	S798C	rs200757344	**0.864**	0.8	
682	p.Met828Val	M799V	rs890006891	0.001	0.4	
683	p.Met828Thr	M799T	rs13306091	0.072	8.0	
684	p.Glu830Lys	E801K	rs267604983	**0.508**	0.8	
685	p.Thr831Ile	T802I	rs777098855	**0.482**	0.4	
686	p.Pro832Leu	P803L	rs761838241	**0.752**	0.4	
687	p.Ser833Phe	S804F	rs1568043397	0.006	0.4	
688	p.Glu835Gly	E806G	rs2030188578	0.998	0.4	
689	p.Gln836Pro	Q807P	rs1599149424	0.006	**50**	Korean
690	p.Leu838Val	L809V	rs2030189487	0.073	0.4	
691	p.Arg840Trp	R811W	rs3730036; (*29*)	**0.612**	281	
692	p.Arg840Gln	R811Q	rs767425642	0.002	3.2	
693	p.Leu841Phe	L812F	rs2030190997	0.293	0.4	
694	p.Phe842Ser	F813S	rs537201274	0.365	0.4	
695	p.Gln843Pro	Q814P	rs1314869920	0.239	0.8	
696	p.Leu848Val	L819V	rs1263864253	0.968	0.8	
697	p.Tyr849Cys	Y820C	rs2030193846	0.998	0.4	

698	p.Asn851His	N822H	rs1599149517	**0.674**	110	Korean
699	p.His853Tyr	H824Y	rs377172559	0.999	0.4	
700	p.Ala854Asp	A825D	rs1258657289	0.999	0.4	
701	p.Ala854Thr	A825T	rs2030195429	0.999	0.4	
702	p.Tyr855Cys	Y826C	rs1290778035	0.997	3.0	
703	p.Val856Met	V827M	rs369111551	0.936	2.8	
704	p.Arg857His	R828H	rs146089353; (*2,10*)	1.000	3.2	Low (*2*)
705	p.Arg857Cys	R828C	rs989791368	1.000	0.8	
706	p.Arg858Gln	R829Q	rs765246562	0.997	**12**	
707	p.Arg858Trp	R829W	rs762333619	1.000	1.2	
708	p.Ala859Thr	A830T	rs1474446784	**0.672**	0.4	
709	p.Leu860Val	L831V	rs1164595381	0.994	0.4	
710	p.Leu860Pro	L831P	rs773277069	1.000	1.7	
711	p.His861Tyr	H832Y	rs140056206; (*7*)	0.006	5.6	
712	p.Arg862Cys	R833C	rs751363862	**0.850**	1.6	
713	p.Arg862His	R833H	rs756018518	0.010	3.6	
714	p.Gly865Arg	G836R	rs939884644	1.000	2.0	
715	p.Ala866Ser	A837S	rs1037068942	0.004	1.5	
716	p.His868Gln	H839Q	rs753727679	0.106	0.0	
717	p.Ile869Val	I840V	rs757164151	0.013	0.8	
718	p.Leu871Gln	L842Q	rs1272138201	0.999	0.4	
719	p.Pro876Ser	P847S	rs757874491	0.999	1.2	
720	p.Ala877Gly	A848G	rs931392712	0.996	0.4	
721	p.His878Gln	H849Q	rs200196657	0.985	1.2	
722	p.Gly881Glu	G852E	rs1232536510	1.000	0.8	
723	p.Asn882Ser	N853S	rs771162255	0.997	0.8	
724	p.Met883Val	M854V	rs774518339	0.994	0.4	
725	p.Met883Thr	M854T	rs1267969615; (*26*)	1.000	0.4	
726	p.Met883Ile	M854L	rs759679121	0.996	4.1	
727	p.Tyr884Arg	Y855R	rs568842388	1.000	3.6	
728	p.Ala885Pro	A856P	rs1173972262	0.071	0.4	
729	p.Ala885Val	A856V	rs1251602237	**0.803**	0.4	
730	p.Thr887Ala	T858A	rs1181835738	0.157	1.1	
731	p.Thr887Asn	T858N	rs761719023	**0.752**	0.4	
732	p.Trp888Ser	W859S	rs1468320560	1.000	0.4	
733	p.Trp888Cys	W859C	rs1157631466	1.000	0.4	
734	p.Tyr892Cys	Y863C	rs750406199	0.993	0.4	
735	p.Val895Leu	V866L	rs1393589215	0.048	0.4	
736	p.Val896Met	V867M	rs145422285	0.039	0.4	
737	p.Pro899Leu	P870L	rs2030335337	0.984	0.4	
738	p.Ala901Ser	A872S	rs752266791	**0.561**	2.4	
739	p.Ser903Trp	S874W	rs558504919	0.429	3.4	
740	p.Met904Thr	M875T	rs779560946	0.029	0.4	
741	p.Asp905Gly	D876G	rs745987517	0.973	1.1	
742	p.Thr906Ala	T877A	rs1274588146	0.000	0.7	
743	p.Thr906Ile	T877I	rs772024137	0.007	0.8	
744	p.Glu908Lys	E879K	rs747159428	0.117	2.0	
745	p.Ala909Ser	A880S	rs1290624816	0.126	0.4	
746	p.Lys912Arg	K883R	rs1329922107	0.002	2.1	
747	p.Gln913Glu	Q884E	rs372614913	0.092	0.4	
748	p.Gln913Arg	Q884R	rs1026599078	0.008	2.4	
749	p.Gly914Asp	G885D	rs1362563545	**0.481**	0.4	

750	p.Trp915Ser	W886S	rs1271898535	0.989	0.7		
751	p.Thr916Ala	T887A	rs755053417	0.422	0.4		
752	p.Thr916Met	T887M	rs3730043; (*7*,*26*,*28*)	0.969	397		AD (*7*)
753	p.Pro917Ala	P888A	rs748317639	0.070	0.4		
754	p.Pro917Arg	P888R	rs770741758	0.989	0.8		
755	p.Met920Thr	M891T	rs745827618	0.948	0.4		
756	p.Lys922Glu	K893E	rs551723440	0.000	0.4		
757	p.Ser930Phe	S901F	rs1231385013	1.000	0.4		
758	p.Leu931Pro	L902P	rs1317192622	1.000	0.4		
759	p.Gly932Arg	G903R	rs1335323894	1.000	2.1		
760	p.Gly932Ala	G903A	rs768235439	0.998	0.4		
761	p.Pro935Ser	P906S	rs199555061	0.922	1.2		
762	p.Pro935Leu	P906L	rs537884559	0.984	8.0		
763	p.Val936Met	V907M	rs752081336	0.036	2.4		
764	p.Glu939Gln	E910Q	rs755034079	0.382	0.7		
765	p.Trp941Arg	W912R	rs375020796	1.000	0.4		
766	p.Trp941Cys	W912C	rs1202055050	0.999	0.4		
767	p.Asn942His	N913H	rs1290388486	0.000	0.4		
768	p.Asn942Lys	N913K	rs201517271	0.003	0.4		
769	p.Asn942Ser	N913S	rs2030363936	0.000	0.4		
770	p.Lys943Arg	K914R	rs777955033	**0.492**	0.8		
771	p.Lys943Asn	K914N	rs1456982290	**0.724**	0.4		
772	p.Leu946Val	L917V	rs1397259349	0.198	0.4		
773	p.Glu947Lys	E918K	rs201076681	**0.902**	1.6		
774	p.Pro949Ser	P920S	rs779881202	0.999	0.4		
775	p.Asp951Tyr	D922Y	rs776223808	0.992	0.8		
776	p.Gly952Arg	G923R	rs987787902	0.993	3.0		
777	p.Arg953Trp	R924W	rs772888815	1.000	1.2		
778	p.Cys957Arg	C928R	rs759966983	0.999	0.4		
779	p.Cys957Ser	C928S	rs767594429	0.994	0.4		
780	p.Ala959Thr	A930T	rs756159839	**0.810**	0.8		
781	p.Ala959Asp	A930D	rs764275894	**0.890**	0.8		
782	p.Ser960Leu	S931L	rs1420430019	0.999	1.6		
783	p.Ala961Pro	A932P	rs779833433	0.999	2.8		
784	p.Asn966Asp	N937D	rs746934582	0.997	0.4		
785	p.Gly967Ser	G938S	rs937878555	0.967	1.6		
786	p.Asp969Tyr	D940Y	rs913463914	0.999	2.1		
787	p.Phe970Cys	F941C	rs2030374256	1.000	0.4		
788	p.Arg971Trp	R942W	rs769406157	1.000	2.4		
789	p.Arg971Gln	R942Q	rs554004241	0.998	8.0		
790	p.Ile972Val	I943V	rs2030513571	0.970	0.8		
791	p.Ile972Thr	I943T	rs2030513778	0.999	0.4		
792	p.Gln974Arg	Q945R	rs375442845	0.995	0.4		
793	p.Cys975Gly	C946G	rs1318768216	1.000	0.4		
794	p.Cys975Tyr	C946Y	rs1346738730	1.000	0.4		
795	p.Thr977Ile	T948I	rs1439040577	0.006	1.1		
796	p.Val978Met	V949M	rs141750591	0.993	**26**		
797	p.Leu980Ser	L951S	rs2030516614	0.266	0.4		
798	p.Val985Met	V956M	rs2030518300	**0.568**	0.7		
799	p.Ala986Pro	A957P	rs1218838386	**0.492**	330	Korean	
800	p.His987Tyr	H958Y	rs1285477249	0.999	1.2		
801	p.His988Arg	H959R	rs1315238107	**0.792**	0.4		

802	p.Glu989Lys	E960K	rs752685131	1.000	1.2	
803	p.Met990Arg	M961M	rs1468555557	**0.601**	0.4	
804	p.Met990Ile	M961I	rs1227030637	0.005	0.8	
805	p.Gly991Ser	G962S	rs756019276	1.000	0.4	
806	p.His992Asp	H963D	rs1244440863	1.000	1.2	
807	p.His992Arg	H963R	rs771384705	0.999	0.8	
808	p.Ile993Val	I964V	rs753450698	0.003	1.4	
809	p.Gln994Arg	Q965R	rs2030522217	0.995	0.4	
810	p.Tyr995His	Y966H	rs778562737	0.999	0.4	
811	p.Phe996Ser	F967S	rs2030522633	0.997	0.7	
812	p.Met997Val	M968V	rs745486055	0.341	1.1	
813	p.Met997Ile	M968I	rs1395070641	0.341	0.4	
814	p.Gln998Lys	Q969K	rs772433710	0.991	0.4	
815	p.Tyr999Cys	Y970C	rs1325658187	1.000	0.4	
816	p.Pro1003Leu	P974L	rs377280373	0.909	2.4	
817	p.Arg1007Lys	R978K	rs747362596	0.999	0.4	
818	p.Gly1009Val	G980V	rs769272334	0.994	2.9	
819	p.Ala1010Gly	A981G	rs1740970700	0.992	0.7	
820	p.Gly1013Ser	G984S	rs571848794; (*7,26*)	1.000	6.8	
821	p.Gly1013Ala	G984A	rs540734174	1.000	0.8	
822	p.His1015Tyr	H986Y	rs2030527371	0.999	0.4	
823	p.His1015Arg	H986R	rs773600140	0.999	1.6	
824	p.His1015Gln	H986Q	rs144751624	0.999	**19**	
825	p.Glu1016Gly	E987G	rs2030528115	1.000	0.4	
826	p.Ala1017Thr	A988T	rs1471502216	0.999	0.4	
827	p.Ile1018Val	I989V	rs2030528735	0.215	0.7	
828	p.Ile1018Thr	I989T	rs4976; (*7*)	0.988	143	
829	p.Gly1019Arg	G990R	rs1418273122	1.000	0.4	
830	p.Asp1020Gly	D991G	rs1200211350	1.000	3.2	
831	p.Val1021Met	V992M	rs764129854	0.977	3.2	
832	p.Leu1024Phe	L995F	rs753672462	1.000	1.6	
833	p.Ser1025Ala	S996A	rs1489092015	0.997	0.7	
834	p.Val1026Met	V997M	rs377550847	0.998	1.2	
835	p.Val1026Ala	V997A	rs1372691116	**0.886**	0.4	
836	p.Ser1027Cys	S998C	rs2030532177	0.980	0.7	
837	p.Thr1028Met	T999M	rs778331848	1.000	2.9	
838	p.Pro1029Ser	P1000S	rs758105347	0.999	0.4	
839	p.Lys1030Asn	K1001N	rs374679629	**0.561**	1.2	
840	p.His1033Pro	H1004P	rs747442787	0.437	0.8	
841	p.Asp1036Lys	N1007K	rs142947404; (*7,28*)	0.041	**71**	
842	p.Leu1037Pro	L1008P	rs1258035065	1.000	0.4	
843	p.Ser1039Asn	S1010N	rs1368193999	0.011	0.8	
844	p.Ser1039Arg	S1010R	rs2030536211	0.034	0.4	
845	p.Ser1040Thr	S1011T	rs749362077	0.000	0.4	
846	p.Glu1041Asp	E1012D	rs771342124	0.000	1.1	
847	p.Gly1043Ser	G1014S	rs2030536853	0.001	0.7	
848	p.Gly1043Val	G1014V	rs1599154510	0.002	0.7	
849	p.Asp1045His	D1016H	rs140980792	0.001	4.8	
850	p.Asp1045Glu	D1016E	rs200011052	0.000	0.8	
851	p.Glu1046Lys	E1017K	rs761601299	0.996	0.4	
852	p.Asp1048Gly	D1019G	rs1313627969	**0.509**	0.8	
853	p.Ile1049Val	I1020V	rs765835019	0.984	0.8	

854	p.Asn1050Asp	N1021D	rs751226904	0.998	0.4	
855	p.Asn1050Ser	N1021S	rs935304784	0.994	1.5	
856	p.Leu1052Val	L1023V	rs989584821	0.988	0.4	
857	p.Leu1052Pro	L1023PV	rs1351048530	1.000	0.4	
858	p.Met1055Leu	M1026L	rs144926742	0.065	4.4	
859	p.Met1055Thr	M1026T	rs1568046795	0.962	0.4	
860	p.Met1055Iso	M1026I	rs767184799	0.017	0.4	
861	p.Ala1056Thr	A1027T	rs569898686	0.999	0.4	
862	p.Asp1058Asn	D1029N	rs1197014458	**0.830**	0.4	
863	p.Asp1058Gly	D1029G	rs1458584759	0.916	0.7	
864	p.Iso1060Val	I1031V	rs1458035301	0.021	1.6	
865	p.Ala1061Thr	A1032T	rs773695336	0.057	4.2	
866	p.Phe1062Leu	F1033L	rs778802598	0.998	0.8	
867	p.Pro1064Leu	P1035L	rs1384728709	**0.671**	1.5	
868	p.Tyr1067His	Y1038H	rs1342469069	0.990	1.1	
869	p.Tyr1067Cys	Y1038C	rs1382876528	0.992	0.7	
870	p.Val1069Iso	V1040I	rs147763588	0.002	2.4	
871	p.Val1069Ala	V1040A	rs1322398043	0.422	3.2	
872	p.Asp1070Asn	D1041N	rs571910640	1.000	4.0	
873	p.Gln1071Lys	Q1042K	rs769531227	0.444	0.4	
874	p.Arg1073Cys	R1044C	rs762807750	1.000	1.6	
875	p.Arg1073His	R1044H	rs141139841	0.999	0.8	
876	p.Val1076Leu	V1047L	rs534480370	0.995	**17**	
877	p.Asp1078Asn	D1049N	rs2030566841	0.338	**18**	Japan
878	p.Asp1078Gly	D1049G	rs375039288	0.946	2.4	
879	p.Gly1079Glu	G1050E	rs752369560	**0.856**	0.8	
880	p.Ser1080Cys	S1051C	rs1472584106	**0.778**	0,4	
881	p.Ser1080Asn	S1051N	rs1166034708	0.111	0.8	
882	p.Iso1081Val	I1052V	rs761139845	0.014	0. 8	
883	p.Iso1081Asn	I1052N	rs2030568517	0.994	0.4	
884	p.Glu1084Lys	E1055K	rs1455896148	0.012	0.8	
885	p.Glu1084Asp	E1055D	rs2030568933	0.001	0.4	
886	p.Gln1088His	Q1059H	rs764489358	**0.874**	0.8	
887	p.Trp1091Arg	W1062R	rs2030569963	1.000	0.7	
888	p.Ser1092Gly	S1063G	rs2030570388	0.082	0.7	
889	p.Arg1094Gly	R1065G	rs754412363	1.000	1.2	
890	p.Gln1098Arg	Q1069R	rs 1568047250; (*30*)	1.000	1.6	36 (*30*)
891	p.Pro1102Thr	P1073T	rs145349565	0.989	**69**	
892	p.Pro1103Thr	P1074T	rs745776314	1.000	5.6	
893	p.Val1104Ala	V1075A	rs1458829834	0.040	0.7	
894	p.Arg1106Ser	R1077S	rs2030598589	1.000	0.4	
895	p.Gln1108Arg	Q1079R	rs2030599332	0.006	0.7	
896	p.Gly1109Val	G1080V	rs768468260	**0.476**	0.7	
897	p.Phe1111Leu	F1082L	rs1465879181	0.996	0.4	
898	p.Asp1112Gly	D1083G	rs1568047328	1.000	0.4	
899	p.Asp1112Glu	D1083E	rs762107963	0.994	0.8	
900	p.Ala1115Asp	A1086D	rs565263717	**0.887**	0.8	
901	p.His1118Tyr	H1089Y	rs1192509168	0.999	0.4	
902	p.Val1123Met	V1094M	rs373319603	0.987	1.2	
903	p.Pro1124Thr	P1096T	rs1455545095	1.000	0.4	
904	p.Iso1126Leu	I1097L	rs567659245	0.022	**11**	
905	p.Iso1126Ser	I1097S	rs2030603816	0.974	0.4	

906	p.Arg1127Ser	R1098S	rs1229959665	0.999	0.7	
907	p.Tyr1128Cys	Y1099C	rs1481913226	1.000	0.4	
908	p.Val1130Iso	V1101I	rs575830312	0.031	2.4	
909	p.Val1130Ala	V1101A	rs2030731929	**0.877**	0.4	
910	p.Ser1131Pro	S1102P	rs777258874	0.998	0.4	
911	p.Ser1131Gly	S1102G	rs2030732506	0.091	0.8	
912	p.Phe1132Leu	F1123L	rs1308790992	0.997	0.8	
913	p.Gln1135Lys	Q1106K	rs749711522; (*26*)	0.995	4.0	
914	p.Gln1135Arg	Q1106R	rs771232505	0.998	0.4	
915	p.Gln1137Arg	Q1108R	rs1895643705	1.000	0.4	
916	p.His1139Tyr	H1110Y	rs1281978641	0.993	0.8	
917	p.Glu1140Lys	E1111K	rs1228691156	0.011	1.2	
918	p.Gln1144Pro	Q1115P	rs775709106	0.063	0.4	
919	p.Ala1145Val	A1116V	rs1444132860	0.032	0.4	
920	p.Ala1146Pro	A1117P	rs2030736169	0.998	0.4	
921	p.His1148Tyr	H1119Y	rs1246627721	**0.900**	0.7	
922	p.Thr1149Met	T1120M	rs764430271	0.439	3.2	
923	p.Gly1150Ala	G1121A	rs762872915	0.439	1.2	
924	p.Pro1151Leu	P1122L	rs1460629643	**0.680**	0.7	
925	p.His1153Gln	H1124Q	rs1167765854	**0.899**	0.4	
926	p.Cys1155Tyr	C1126Y	rs1319412351	0.999	1.9	
927	p.Asp1156Arg	D1127R	rs751557067	0.998	0.4	
928	p.Ile1157Thr	I1128T	rs1215104945	0.999	2.4	
929	p.Ser1160Phe	S1131F	rs1435131111	1.000	0.4	
930	p.Glu1162Asp	E1133D	rs2030740252	0.006	0.4	
931	p.Ala1163Ser	A1134S	rs1299150514	0.998	1.2	
932	p.Gly1164Arg	G1135R	rs145579007	1.000	**10**	
933	p.Arg1166Cys	R1137C	rs777499791	**0.456**	1.2	
934	p.Arg1166His	R1137H	rs201126192	0.273	8.0	
935	p.Leu1167Val	L1138V	rs1227093005	0.994	1.4	
936	p.Leu1167Pro	L1138P	rs2030743196	1.000	0.4	
937	p.Ala1168Thr	A1139T	rs771285769	0.439	0.4	
938	p.Ala1168Val	A1139V	rs993733529	**0.647**	0.7	
939	p.Ala1170Thr	A1141T	rs747412511	**0.756**	0.8	
940	p.Ala1170Val	A1141V	rs2030766252	0.058	0,7	
941	p.Met1171Leu	M1142L	rs776634959	0.018	0.4	
942	p.Gly1174Asp	G1145D	rs1458534207	1.000	1.1	
943	p.Arg1177Ser	R1177S	rs1385800001	0.036	0.8	
944	p.Pro1178Leu	P1149L	rs538659872	**0.836**	1.6	
945	p.Pro1180Ala	P1151A	rs767177049	0.995	2.0	
946	p.Pro1180Leu	P1151L	rs775501006	1.000	5.6	
947	p.Met1183Val	M1154V	rs376826294	0.957	0.8	
948	p.Met1183Thr	M1154T	rs753388395	**0.634**	0.4	
949	p.Met1183Ile	M1154I	rs756739847	0.998	0.4	
950	p.Iso1186Met	I1157M	rs764893306	0.059	0.8	
951	p.Thr1187Met	T1158M	rs12709442	1.000	**76**	
952	p.Gln1189Arg	Q1160R	rs1210452594	0.995	0.8	

953	p.Pro1190Ser	P1161S	rs1295931503	0.028	1.9	
954	p.Pro1190Leu	P1161L	rs1264163829	**0.663**	0.7	
955	p.Met1192Val	M1163V	rs2030771924	**0.654**	6.0	
956	p.Ser1193Asn	S1164N	rs780228220	0.996	1.6	
957	p.Ala1194Thr	A1165T	rs755506668	**0.898**	0.8	
958	p.Ala1194Asp	A1165D	rs777388821	0.982	0.4	
959	p.Ser1195Pro	S1166P	rs1343107612	0.092	0.4	
960	p.Ser1195Leu	S1166L	rs748284095	0.004	2.4	
961	p.Ala1196Thr	A1167T	rs773596097	0.433	0.4	
962	p.Met1197Val	M1168V	rs201870045	0.016	1.2	
963	p.Phe1201Leu	F1172L	rs775204602	0.998	0.4	
964	p.Pro1203Leu	P1174L	rs369760270	1.000	1.6	
965	p.Asp1206Tyr	D1177Y	rs2030775874	**0.871**	0.4	
966	p.Asp1206Ala	D1177A	rs1291258688	0.164	0.8	
967	p.Arg1209Pro	R1180P	rs5381166970; (*2,10*)	0.358	1.6	203* (*10*)
968	p.Arg1209His	R1180H		0.240		
969	p.Arg1209Cys	R1180C	rs1353694784	0.414	0.8	
970	p.Thr1210Met	T1181M	rs12720742	0.421	106	
971	p.Glu1211Lys	E1182K	rs766053859	0.116	0.9	
972	p.Glu1211Val	E1182V	rs1568049110	**0.880**	0.4	
973	p.Asn1212Asp	N1183D	rs996589954	0.999	1.4	
974	p.Asn1212Thr	N1183T	rs574717474	1.000	2.4	
975	p.Glu1213Lys	E1184K	rs149590791	0.001	0.4	
976	p.Glu1213Ala	E1184A	rs2030780027	0.001	0.7	
977	p.Leu1214Pro	L1185P	rs1451027689	0.058	1.1	
978	p.His1215Arg	H1186R	rs757974064	0.040	0.4	
979	p.His1215Gln	H1186Q	rs144312383	0.003	6.0	
980	p.Gly1216Arg	G1187R	rs2030781426	**0.699**	0.4	
981	p.Glu1217Asp	E1188S	rs1248102885	0.048	0.4	
982	p.Lys1218Gln	K1189Q	rs2030782089	0.001	0.4	
983	p.Lys1218Arg	K1189R	ss2030782285	0.023	0.4	
984	p.Leu1219Pro	L1190P	rs140941300	0.989	4.7	
985	p.Gly1220Ser	G1191S	rs1418538736	1.000	0.4	
986	p.Pro1222Ser	P1193S	rs144888208	0.999	0.4	
987	p.Pro1222Leu	P1193L	rs779175881	1.000	2.4	
988	p.Tyr1224His	Y1195H	rs776554544	0.962	0.4	
989	p.Asn1225Lys	N1196K	rs1033103629; (*15*)	0.137	2.4	**434** (*15*)
990	p.Asn1225Thr	N1196T	rs761685671	0.001	0.4	
991	p.Trp1226Cys	W1197C	rs769710002	1.000	0.4	
992	p.Thr1227Ala	T1198A	rs772778762	0.000	0.8	
993	p.Thr1227Met	T1198M	rs762495578	0.010	4.5	
994	p.Pro1228Leu	P1199L	rs121912703; (*31*)	1.000	3.7[d]	**425** (*14,31*)
995	p.Asn1229Ser	N1200S	rs753269825	0.000	0.4	
996	p.Ser1230Tyr	S1201Y	rs756742824	0.372	2.9	
997	p.Ala1231Thr	A1202T	rs959741765	0.183	0.4	
998	p.Arg1232Cys	R1203C	rs750545791	0.265	1.1	

999	p.Arg1232His	R1203H	rs372282664; (*7*)	0.001	6.9		
1000	p.Ser1233Leu	S1204L	rs1474601688	0.001	6.0		
1001	p.Pro1236Leu	P1207L	rs751737727	0.001	0.4		
1002	p.Asp1239Gly	D1210G	rs777561376	0.000	0.9		
1003	p.Asp1239Glu	D1210E	rs749019292	0.000	0.4		
1004	p.Gly1241Ser	G1212S	rs367916721	0.003	**38**		
1005	p.Arg1242Cys	R1213C	rs1226490350	**0.446**	1.4		
1006	p.Arg1242His	R1213H	rs781198085	0.374	3.5		
1007	p.Val243Ile	V1214I	rs372416620	0.279	5.6		
1008	p.Phe1245Leu	F1216L	rs521181910	0.039	0.4		
1009	p.Gly1247Val	G1218V	rs2030812434	0.976	0.4		
1010	p.Asp1249Ala	D1220A	rs777124668	0.001	0.4		
1011	p.Leu1250Val	L1221V	rs1193002337	0.034	0.4		
1012	p.Ala1252Val	A1223V	rs762056936	0.007	0.4		
1013	p.Arg1256Cys	R1227C	rs763049172	0.353	**32**		
1014	p.Arg1256His	R1227H	rs766377685	0.001	1.8		
1015	p.Val1257Met	V1228M	rs759857038	0.016	0.5		
1016	p.Gly1258Ser	G1229S	rs1347084405	0.341	0.5		
1017	p.Gly1258Asp	G1229D	rs1434928128	**0.888**	0.8		
1018	p.Gln1259Pro	Q1230P	rs756978461	**0.497**	0.5		
1019	p.Gly1266Asp	G1237S	rs778929965	0.075	5.3		
1020	p.Ala1268Thr	A1239T	rs757997489	0.005	3.4		
1021	p.Leu1270Pro	L1241P	rs1179455074	0.024	0.8		
1022	p.Val1271I	V1242I	rs780690514	0.003	0.4		
1023	p.Ala1272Thr	A1243T	rs1425238764	0.029	0.8		
1024	p.Ala1272Gly	A1243G	rs770016471	**0. 530**	1.4		
1025	p.Arg1279Gln	R1250Q	rs4980; (*7* , *28*)	0.002	410	85 (*4*)	AD (*28* , ♀)
1026	p.Arg1279Trp	R1250W	rs568401628	**0.451**	4.5		
1027	p.Phe1281Ser	F1252S	rs1393052928	0.004	0.7		
1028	p.Iso1283Val	I1254V	rs1268051765	0.011	0.8		
1029	p.Arg1284Cys	R1255C	rs375527470; (*7*)	0.353	5.4		
1030	p.Arg1284His	R1255H	rs1333987355	0.001	0.7		
1031	p.Arg1286Ser	R1257S	rs4364; (*20* ,*28*)	0.013	733		
1032	p.Arg1286Cys	R1257C		**0.733**			
1033	p.Arg1286His	R1257H	rs767828019	0.000	**17**		
1034	p.Leu1288Phe	L1259F	rs2030824754	0.008	0.4		
1035	p.His1289Arg	H1260R	rs1004296792	0.000	1.1		
1036	p.Arg1290Trp	R1261W	rs752812293	0.000	**42**		
1037	p.Arg1290Gln	R1261Q	rs12720745	0.000	622		
1038	p.His1291Pro	H1262P	rs2030827530	0.000	0.4		
1039	p.His1293Tyr	H1264Y	rs765069550	0.027	6.0		
1040	p.His1293Gln	H1264Q	rs1013454628	0.001	4.0		
1041	p.Gly1294Arg	G1265R	rs1422356094	0.006	0.4		
1042	p.Gly1294Glu	G1265E	rs2030827530	0.004	0.7		
1043	p.Pro1295Leu	P1266L	rs886053226	**0.466**	0.8		
1044	p.Gln1296Arg	Q1267R	rs4961	0.001	0.7		

1045	p.Gly1298Cys	G1269C	rs1024799181	0.009	2.9	
1046	p.Glu1300Lys	E1271K	rs751134637	0.313	2.8	
1047	p.Glu1300Gly	E1271G	rs1385347177	**0.725**	0.7	
1048	p.Glu1300Asp	E1271D	rs1244045213	**0.453**	0.8	
**III. Combined frequency**						
**Probably damaging (red)**					**3,854**	
**Possibly damaging (violet)**					**1,700**	
**Probably damaging and possibly damaging combined**					**5,554**	
**Benign**					**4,871**	
**All (MAF)**					**10,425**	

Name of the ACE mutations (column C) , that were already phenotyped for blood ACE levels (column G), were marked with red.
[a] Japanese; [b]Mutations, eliminating transmembrane anchor, i.e. increasing (>10-fold) blood ACE, and thus, decreasing tissue ACE; [c]mostly African; [d] much more frequent in Netherlands [18];
Frequency of mutations (MAF, column 6): >10-**bold**, >100-red, >1000-bold **red.**
Blood ACE levels (column G) is a median for several carriers of given mutation and expressed as % of mean in population, corrected for genotype (I/D polymorphism).

Polyphen2: PolyPhen 2 (dbNSFP version 3.3a) annotation based on HumanVar database. This annotation should be used when evaluating rare alleles at loci potentially involved in complex phenotypes, dense mapping of regions identified by genome-wide association studies, and analysis of natural selection from sequence data. The annotation consists of score and categorical prediction. There are three possible predictions: **D** (Probably damaging, score>=0.909), **P** (possibly damaging, **0.446**<=score<=**0.908**), B (benign,

ACE mutations analysed in this study

Table S2. ACE mutations with measured blood ACE   Blood ACE: **62**   Total: **1234**   9/29/23

#	Genetic position	Amino acid position (mature protein)	Polymorphism or (*reference*)	PolyPhen-2 Score (HVAR)	Minor Allele Frequency, per 100 000	Blood ACE, % of M	
**I. Damaging (elimination ?) of signal peptide (SP)**							
7	p.Ser5GlyfsX136	SP	rs797045079; (*1*)	1.000	0.4	Low	
15	p.Arg8GlyfsX134	SP	(*2*)	1.000	0.4	Low	
23	p.Leu13_Leu14del	SP	rs900084108; (*2*)	1.000	6.6	Low	
24	p.Leu13_Leu16del	SP	rs751352152; (*3*)	1.000	0.8	Low	
32	p.Leu16_Pro23indel	SP	rs983649759; (*2*)	1.000	**19**	Low	
36	p.Leu18_L20ins	SP	rs532691783; (*4*)	1.000	6.0	86 (*4*)	
41	p.Leu21Pro	SP	(*2*)	**0.797**	0.4	Low	
**II. Indels or stop codons in mature ACE**							
9	p.Arg149Leufs*54	R120LfsX54	rs778759606; (*2,5*)	1.000	4.2	Low	AD (*5*)
19	p.Arg265X	R236X	rs138873311; (*2*)	1.000	1.2	Low	
20	p.Tyr266X	Y237X	rs121912704; (*6,7*)	1.000	0.8	Low	
23	p.Arg274GlyfsX117	R245Gfs	(*8*)	1.000	0.4	Low	
29	p.Glu328del	E299del	(*2*)	1.000	0.4	Low	
31	p.Trp343X	W314X	rs200225958; (*2,5*)	1.000	0.8	Low	AD (*5*)
32	p.Ser346GlufsX47	S317Efs	rs1331062614; (*2*)	1.000	0.4	Low	
39	p.Leu440ProfsX15	L411Pfs	rs387906576; (*6*)	1.000	0.4	Low	
40	p.Asp441fs	D412fs	(*5*)	1.000	0.4	Low	AD (*5*)
47	p.Pro485Leufs	P456fs	(*9*)	1.000	0.4	Low	
51	p.Arg496X	R467X	rs397514688; (*2*)	1.000	0.4	Low	
55	p.Arg508X	R479X	rs367797185; (*2*)	1.000	3.2	Low	
56	c.1709+5G>T	Abn. splicing	(*2*)	1.000	0.4	Low	
62	p.Trp581Glyfs	W552Gfs	(*2*)	1.000	0.4	Low	
64	p.Lys601AsnfsX40	K572Nfs	(*2,10*)	1.000	0.8	Low	
72	p.Trp672X	W643X	(*11*)	1.000		Low	
75	p.Ile717Glnfs	I688Qfs	rs1219522144; (*2*)	1.000	0.8	Low	
76	p.Ile721LysfsX60	I692Lfs	(*2*)	1.000	0.4	Low	
83	p.Arg791X	R762X	(*2,10*)	1.000	0.4	Low	
85	p.Tyr805X	Y776X	rs761458810	1.000	0.4	49 (personal)	
94	p.Pro897fs	P868fs	(*12*)	1.000	0.4	Low	
101	p.Leu1024fs	L995fs	(*5*)	1.000	0.4	Low	AD (*5*)
103	p.Leu1032fs	L1003fs	(*12*)	1.000	0.4	Low	
105	p.Asp1058Tyrfs	D1029Yfs	(*5*)	1.000	0.4	Low	AD (*5*)
121	p.Gln1165X	Q1136X	(*2,10*)	1.000	0.4	Low	
122	p.Lys1172_Met1183del	K1143_M1154del	(*1*)	1.000	0.4	Low	
123	c.3503+1G>A	Abn. splicing	(*2*)	1.000	0.4	Low	

124	c.3691+1G>A	Abn. splicing	(*13*)	1.000	4.4	1133	
125	p.Gly1174AlsfsX12	G1145Afs	rs754265941;(*2,8*)	1.000	**47**	Low	
129	p.Trp1226X	W1197X[b]	rs769710002; (*14*)	1.000	0.4	1300	
130	p.Ser1238Pfs	S1209Pfs	(*5*)	1.000	0.4	Low	AD (*5*)
131	p.Gln1253X	Q1224X[b]	rs1174820268; (*15*)	1.000	0.4	1200	
**III. All missense mutations (including damaging)**							
14	p.Gly45Arg	G16R	rs750712925	0.999	2.9	Low (*16*)	
181	p.Tyr244Cys	Y215C	rs3730025; (*7,16-19*)	1.000	**1068**	73 (*4,19*)	AD (*17-18*)
195	p.Arg259His	R230H	rs370903033; (*2,9*)	0.995	1.2	Low (*9*)	
222	p.Gln288Arg	Q259R	rs199591851; (*2,9*)	0.998	74	68 (*4*)	
260	p.Leu333Gln	L304Q	rs761390621; (*2*)	1.000	0.7	Low	
275	p.Gly354Arg	G325R	rs56394458; (*7*)	0.998	780	62 (*4*)	
285	p.Ser362Trp	S333W	rs142328237; (*21*)	1.000	6.8	71 (*21*)	
367	p.Arg459Gln	R430Q	(*22*)			Low (*22*)	
379	p.Arg482Cys	R453C	rs201540553; (*7*)	**0.649**	**19**	Low (*16*)	
384	p.Pro485Arg	P456R	rs28730839; (*7*)	0.301	**48**	98 (*4*)	
385	p.Pro485Leu	P456L	(*9*)			Low (*9*)	
396	p.Tyr494Asp	Y465D	rs760325775; (*20,23*)	0.011	2.4	700 (*23*)	
398	p.Arg496Gln	R467Q	rs761345398; (*22,24*)	1.000	1.9	Low (*22*)	
408	p.Pro505Ala	P476A	rs148943954; (*7*)	0.939	**59**	147 (*4*)	
457	p.Arg561Trp	R532W	rs4314; (*7,20,25*)	**0.783**	**78**	500 (*25*)	
516	p.Trp623Arg	W594R	(*2,10*)	1.000	0.4	Low (*2*)	
524	p.Pro630Leu	P601L	rs142818229; (*4*)	0.988	4.1	154 (*4*)	
532	p.Gly639Ser	G610S	rs72845024; (*4*)	0.007	6.1	142 (*4*)	
704	p.Arg857His	R828H	rs146089353; (*2,10*)	1.000	3.2	Low (*2*)	
890	p.Gln1098Arg	Q1069R	rs 1568047250; (*30*)	1.000	1.6	36 (*30*)	
967	p.Arg1209Pro	R1180P	rs5381166970; (*2,10*)	0.358	1.6	203* (*10*)	
989	p.Asn1225Lys	N1196K	rs1033103629; (*15*)	0.137	2.4	434 (*15*)	
994	p.Pro1228Leu	P1199L	rs121912703; (*31*)	1.000	3.7[d]	425 (*14,31*)	More frequent in the Netherlands [31]
1025	p.Arg1279Gln	R1250Q	rs4980; (*7, 28*)	0.002	410	85 (*4*)	AD (*28*, ♀)

Name of the mutations (column 3) that are already confirm as LoF were marked with red

Frequency of mutations (MAF, Column 6): >10-bold; >100-red; > 1000-bold red

PolyPhen-2: PolyPhen 2 (dbNSFP version 3.3a) annotation based on HumanVar database.

There are three possible predictions: D (Probably damaging, score>=0.909),

P (Possibly damaging, **0.446**<=score<=**0.908**), B (benign, score<=0.445)

ACE mutations, analysed in in this study

Blood ACE levels are presented as median of blood ACE levels corrected for genotype of ACE I/D Plymorphism
